# Targeting endothelial MYC using siRNA or miR-218 nanoparticles sensitizes chemo- and immuno-therapies by recapitulating the Notch activation-induced tumor vessel normalization

**DOI:** 10.7150/thno.112023

**Published:** 2025-04-13

**Authors:** Xianchun Yan, Ziyan Yang, Xiuli Cao, Liang Liang, Yanyan Duan, Peiran Zhang, Yixuan Feng, Ting Wen, Shanqiang Luo, Lintao Jia, Jiaxing Sun, Hua Han

**Affiliations:** 1 State Key Laboratory of Holistic Integrative Management of Gastrointestinal Cancers and Department of Biochemistry and Molecular Biology, Fourth Military Medical University, Xi'an 710032, China.; 2 Department of Medical Genetic and Developmental Biology, Fourth Military Medical University, Xi'an, 710032, China.; 3 Department of Ophthalmology, Eye Institute of Chinese PLA, Xijing Hospital, Fourth Military Medical University, Xi'an 710032, China.

**Keywords:** Notch, tumor vessel normalization, nanoparticles, MYC, miR-218

## Abstract

**Background:** The chaotic, over-activated tumor vasculature promotes tumor growth and erodes most current therapies. Although Notch activation critically regulates angiogenesis, the broad roles of Notch has dampened its druggability.

**Methods:** Gene-modified mice with a Cdh5-Cre^ERT^ transgene were employed to activate/block Notch signaling in endothelial cells (ECs). Multiple transcriptome analyses were conducted to compare gene expression profiles. qRT-PCR and western blotting were used to determine gene expression level. Immunofluorescence and flow cytometry were used to observe morphological alterations and immune microenvironment in tumors. Nanoparticles (PEI-PEG-cRGD) were used to deliver siRNA into tumor ECs (TECs) *in vivo*.

**Results:** Genetic Notch activation or blockade in TECs normalizes or deteriorates tumor vessels, respectively. Single-cell RNA sequencing showed that Notch activation selectively reduced the proliferating TEC subset, which accounted for about 30% of TECs and gave rise to other TEC subsets. Notch activation or blockade downregulated or upregulated MYC, respectively. MYC overexpression canceled Notch activation-induced proliferation arrest of TECs *in vitro*, and a MYC inhibitor normalized tumor vessels in RBPj deficient mice, suggesting that MYC is the authentic Notch target in normalizing tumor vessels. Nanoparticles encapsulated with MYC siRNA (EC-siMYC) or miR-218 (EC-miR-218), a Notch-downstream miRNA suppressing MYC, were able to mitigate Notch inhibition-induced tumor vessel defects. Combination of cisplatin with MYC blockade exhibited improved therapeutic effects. Moreover, MYC blockade promoted T cell infiltration and enhanced anti-PD1 immunotherapy.

**Conclusions:** Together, our data have demonstrated that Notch activation normalizes tumor vessels by repressing the proliferating TEC subset via MYC, and targeting endothelial MYC using nanoparticles bearing siRNA or miRNA is an efficient strategy for tumor anti-angiogenic therapy.

## Introduction

To support the fast and continuous proliferation of malignant cells, tumors are destined to develop their neovascular networks by angiogenesis. However, tumor vessels are characteristically tortuous, resulting in stagnant and chaotic blood flow, high interstitial pressure, and hypoxia, which enhance malignancy and therapy resistance 1. Therefore, anti-angiogenic therapies (AATs) have been proposed to correct the structural and functional anomalies of tumor vessels, a therapy termed as vascular normalization 1. Indeed, strategies targeting the vascular endothelial growth factor receptor 2 (VEGFR2) signaling induce vascular normalization and tumor regression in preclinical models and cancer patients when combined with other therapies such as chemo- and immuno-therapies 2, 3. However, the efficacy of current AATs is limited and transitory, accompanied by emerging resistance 4-6, prompting identification of alternative targets and strategies.

Endothelial cells (ECs) are the major host cell population participating in tumor angiogenesis 7-9. Tumor ECs (TECs) undergo abnormal proliferation, differentiation, and fate transitions in response to disordered pro-angiogenic milieu in tumor microenvironment (TME) 10-12. Moreover, as suggested by recent single-cell RNA sequencing (scRNA-seq) studies, TECs are highly heterogeneous with continuous adoptive remodeling of their transcriptomes, blunting current AATs 13-18. Indeed, it has been shown that the VEGF-blocking therapy, the most common AAT currently used in clinics, can target only less than 10% of TECs 17, 18. These facts suggest that characterization of different TEC subsets could provide more efficient strategies for AATs. In addition, to target TECs, different nanomaterial-based delivery tools have been developed 19, 20. For example, the cyclo Arg-Gly-Asp-D-Phe-Lys peptide (cRGD) has been shown to target TECs via integrin αvβ3 21. These materials have provided an opportunity to interfere with intracellular molecules and pathways of TECs precisely with siRNAs or miRNAs, supposing that critical TEC subsets and pathways/molecules are identified.

The Notch-recombination signal binding protein Jkappa (RBPj) pathway widely participates in vessel morphogenesis and regulates EC proliferation and differentiation 22. Endothelial Notch blockade results in over-sprouting and abnormal vessel formation 23-25, while Notch activation restricts angiogenesis by limiting EC activation and remodeling metabolism, leading to reduced tumor growth 26-29. However, Notch signaling is widely involved in development and homeostasis, making it poorly druggable in cancer treatment 3, 6. To access this question, in the current study, we examined TEC subsets in mice with endothelial Notch activation, and found that Notch activation normalized tumor vasculature likely by repressing the proliferating TEC subset via MYC. Based on our previous findings that Notch activation suppresses MYC through miR-218-5p (hereafter referred to as miR-218) and miR-342-5p (hereafter referred to as miR-342) 30-32, we tested targeting MYC with these miRNAs in TECs using EC-targeted nanoparticles. We show that endothelial delivery of MYC siRNA or miR-218 recapitulates Notch activation-induced tumor vessel normalization and remarkably facilitates chemo- and immuno-therapies in mice.

## Materials and Methods

### Human Samples

Human lung cancer paraffin samples (Table S1 in Supplementary Information) were purchased from Outdo Biotech (HLugA030PG04-2, Shanghai, China). The use of human samples was approved by the Ethics Committee of Xijing Hospital and were performed in accordance with the Declaration of Helsinki (KY20194052).

### Animal models

Male C57BL/6 mice were maintained under specific pathogen-free (SPF) conditions. Rosa-Stop^floxed^-*NIC*, Cdh5-Cre^ERT^, and *RBPj*^floxed^ mice were described previously 33-35. Mice were genotyped by PCR using tail DNA and primers listed in Supplementary Table S2. For induction of Cre-mediated recombination, male mice aged 6 - 8 weeks were injected daily with tamoxifen (100 mg/kg, Sigma-Aldrich, St. Louis, MO, USA) intraperitoneally (i.p) for 5 days. All animal experiments were reviewed and approved by the Animal Experiment Administration Committee of the Fourth Military Medical University (IACUC-20190707).

For tumor models, Lewis lung carcinoma (LLC) or melanoma (B16-F10) cells were inoculated subcutaneously (s.c) on the right back of mice (1 × 10^6^ cells/100 μl PBS) next day to the last injection of tamoxifen 35. Tumors were dissected 21 days or 16 days for LLC or B16, respectively, after the inoculation, and tumor weight and size (π × [d2 × D]/6 [d, short diameter, D, long diameter]) were evaluated. Cisplatin (2.5 mg/kg) (MedChem Express, Monmouth Junction, NJ) was injected i.p every two days from day 7 after inoculation depending on experiments. The MYC inhibitor 10058-F4 (30 mg/kg) was purchased from TargetMol (Boston, MA, USA) and administered i.p every other day from day 7 after tumor inoculation. For VEGF signaling blockade, the VEGFR2 inhibitor Ki8751 (20 mg/kg, TagetMol) was injected i.p every other day from day 7 after tumor inoculation 36. For combination treatment with immunotherapy, anti-mouse PD-1 antibody (100 μg, MedChem Express) or saline was administered i.p every 2 days from day 13 after tumor inoculation.

To evaluate metastasis, LLC cells were infected with lentivirus expressing luciferase to construct a stable cell line (LLC-Luc). At day 21 after inoculation with LLC-Luc, tumors were surgically removed after anesthetizing with 1% pentobarbital sodium. On day 21 after tumor resection, mice were intraperitoneally injected with D-luciferin (150 mg/kg, Yesen, Shanghai, China), and sacrificed 7 min later. Lungs were harvested, photographed and analyzed with a bioluminescence imaging system (IVIS) (Xenogen Perkin-Elmer, Fremont, CA).

### Histology

Mice were anesthetized and perfused by intracardiac injection of PBS and their tumors or normal tissues (heart, lung, liver and kidney) were harvested. For immunohistochemistry, tissues were fixed overnight in 4% paraformaldehyde (PFA) at 4 °C, embedded in paraffin wax, and sectioned at 5 μm thickness. H&E staining was performed according to standard protocols. Images were acquired using a microscope.

For immunofluorescence, tissues were fixed in 4% PFA at 4 °C for 4 h, followed by dehydration in 30% sucrose in PBS overnight. Samples were embedded in optimal cutting temperature (OCT) compound (Sakura Finetek, Inc, Torrance, CA), cryosectioned at 8 μm thickness, and dried for 2 h at room temperature. Sections were then blocked with 1% bovine serum albumin (BSA) in PBS, permeabilized with 0.5% Triton X-100, and incubated overnight at 4 °C with primary antibodies. After washing, sections were incubated with secondary antibodies at room temperature for 1 h. Images were captured under a fluorescence or laser-scanning confocal fluorescence microscopes (A1R, Nikon, Shanghai, China). Cell samples were fixed with 4% PFA for 30 min at room temperature, permeabilized with 0.3% Triton X-100, and blocked in 5% BSA for 30 min at room temperature. Samples were stained in the same way and nuclei were counter-stained with Hoechst 33258 (Sigma-Aldrich). Antibodies are listed in Table S3 in Supplementary Information.

For immunofluorescence of human lung cancer samples, sections were stained using tyramide signal amplification (TSA) technology by a commercial service (Servicebio, Wuhan, China). Briefly, tumor sections were dewaxed, repaired with antigen, blocked with 3% BSA in PBS for 30 min, and incubated with the first kind of primary antibody overnight at 4 °C. After washing, secondary antibody was added and incubated. In an hour, sections were washed three times and incubated with corresponding fluorescently labeled TSA for 10 min at room temperature. After washing, tissue sections were re-repaired and the antibodies and fluorescently labeled TSA incubation process followed according to the previous steps. Images were captured under a fluorescence scanner (3DHISTECH, BP, Hungary).

To evaluate hypoxia, tumor-bearing mice were injected with pimonidazole hydrochloride (60 mg/kg) 1 h before tumor harvest. Cryosections were stained with a Hypoxyprobe-1-Mab1 kit (Hypoxyprobe, Inc, Burlington, MA) according to the manufacturer's instructions. In some experiments, hypoxia was evaluated by GLUT1 staining 35. To examine vascular perfusion and permeability, mice were intravenously administered 100 μl FITC-Dextran-2MD or tetramethyl rhodamine isothiocyanate-Dextran-70KD (25 mg/mL) (Sigma-Aldrich). Mice were perfused 15 min after the injection and analyzed by immunofluorescence. In some experiments, vascular perfusion was examined by intravenously injected with dylight 594 labeled lectin (50 μg per mouse) (Vectorlabs, Newark, CA).

### Isolation of TECs

Tumors were minced mechanically and digested in 1 mg/mL collagenase I and 10 μg/mL DNase I (Sigma-Aldrich) for 45 min at 37 °C. After passing through a 70-μm tissue strainer, cell suspensions were centrifuged for 4 min at 1200 rpm at 4 °C, followed by erythrolysis. Cell pellets were resuspended in 90 μl PBS containing 0.5% BSA and 2 mM EDTA and mixed with 10 μl anti-CD31-coated beads (Miltenyi Biotec, Bergisch Gladbach, Germany). After incubation at 4 °C for 30 min, cells were collected using a magnetic bead collector (Miltenyi Biotec) and washed three times with PBS containing 0.5% BSA and 2 mM EDTA. Cells were evaluated by flow cytometry after staining with anti-EMCN and by a diluted acetylated fluorescently labeled low-density lipoprotein (Dil-Ac-LDL) uptake assay kit (H7970, Solarbio, Beijing, China).

### Flow cytometric analysis

LLC tumors were harvested and dissociated in a digestion solution of collagenase I (1 mg/ml, Sigma-Aldrich) and DNase I (100 μg/ml, Sigma-Aldrich) in RPMI 1640 medium at 37 °C. After filtering through a 70-μm cell strainer, red blood cells were lysed using ACK lysis buffer (Beyotime, Shanghai, China). Cells were then diluted in PBS containing 2% inactivated FBS and 0.01% NaN_3_, and stained with panels of the antibodies (Biolegend) for 30 min on ice. For cytoplasmic staining, cells were fixed and permeabilized with a Transcription Factor Staining Buffer Set (eBioscienc, San Diego, CA). Analysis was performed using FACS CantoII^TM^ (BD Pharmingen, San Diego, CA). Data were analyzed using the Flowjo V.10 software (TreeStar). T cell panel: BV-510-CD45, APC-CD3, FITC-CD8, PE-CD4, 7-aminoactinomycin D (7-AAD). Myeloid cell panel: BV510-CD45, FITC-CD11b, APC-F4/80, APC-Cy7-Ly6G, 7-AAD. Primary antibodies are listed in supplementary Table S3.

### Cell culture and transfection

LLC and B16-F10 cells, bEnd.3 mouse brain EC line and A549 lung cancer cells were obtained from American Type Culture Collection (ATCC, Manassas, VA). Cells were maintained in Dulbecco's modified Eagle's medium (DMEM) supplemented with 10% fetal calf serum (FCS) and 2 mM L-glutamine (Invitrogen, Carlsbad, CA). Human umbilical endothelial cells (HUVECs) were cultured in EC medium (ScienCell, San Diego, CA) supplemented with 5% fetal bovine serum (FBS) (Invitrogen) and EC growth supplements (ECGS) (ScienCell) and used in experiments between passages 3 and 5 as previous described 30.

For Notch activation, adenovirus expressing human Notch1 ICD (AdNIC, 5261-7665 bp from the first cDNA nucleotide, NM_017617.4) was purchased from Hanbio Biotechnology (Shanghai, China) and used at multiplicity of infection (MOI) of 50 according to the operation procedures. For Notch signal blockade, DAPT (Selleck Chemicals, Houston, TX), a γ-secretase inhibitor, was used at concentration of 25 μM with dimethyl sulfoxide (DMSO) as a control. For MYC overexpression, HUVECs were infected with an adenovirus expressing MYC or control (HanBio) at a MOI of 100 as previously described 30, 32. For MYC blockade *in vitro*, bEnd.3 cells were transfected with 100 nM siRNA to MYC (siMYC, GenePharma) or control oligonucleotide using Lipofectamine 2000^TM^ (Invitrogen). The cells were collected 24 or 48 h after transfection. 10058-F4 was used at the final concentration of 10 μM with DMSO as a control.

A549 tumor cell derived conditioned medium (TCM) was prepared as previous described 35. Briefly, tumor cells were inoculated and cultured in 10-cm culture dishes for 12 h. The medium was replaced with 10 mL serum-free medium (SFM). For 48 h, the medium was harvested, filtered and centrifuged. The TCM or SFM was mixed 1:1 with ECM for subsequently application.

### Cell proliferation assay

Cell proliferation *in vitro* was evaluated using a 5-Ethynyl-2'-deoxyuridine (EdU) incorporation assay according to the standard procedure. Cells were treated with EdU (50 μM, RiboBio, Guangzhou, China) and incubated for 2 h. Cells were then fixed with 4% PFA for 20 min at room temperature, and stained with Apollo 567 and Hoechst. Images were captured under a fluorescence microscope. Proliferation ability of ECs was determined as the ratio of EdU^+^ cells in total Hoechst^+^ cells.

### Bulk RNA sequencing (RNA-seq)

TECs from LLC tumors from three pairs of NIC^eCA^ and control mice on day 14 after inoculation were subjected to RNA extraction using TRIzol. RNA integrity was evaluated using an Agilent 2200 Tape Station (Agilent Technologies, Santa Clara, CA) and each sample had RNA Integrity Numbers (RINes) above 7.0. Ribosomal RNA (rRNA) was removed using the Epicentre Ribo-Zero rRNA Removal Kit (Illumina, San Diego, CA). The remaining RNA was fragmented into ~200 bp fragments. Subsequently, the samples were subjected to first and second-strand cDNA synthesis followed by adaptor ligation and enrichment with low-cycle PCR using a TruSeq® RNA LT/HT Sample Prep Kit (Illumina). The purified library products were evaluated using the Agilent 2200 TapeStation and Qubit®2.0 (Life Technologies, Foster City, CA) and then diluted to 10 pM for cluster generation *in situ* on a HiSeq3000 pair-end flow cell followed by sequencing (2 × 150 bp) on a HiSeq 3000 (RiboBio, Guangzhou, China). Bioinformatic analysis was performed using the OmicShare tools at www.omicshare.com/tools.

### Single cell RNA sequencing (scRNA-seq)

For scRNA-seq, TEC suspensions (~10,000 cells) were loaded on the Chromium Single Cell Instrument (10× Genomics, San Francisco, CA) and converted to barcoded scRNA-seq libraries using a Chromium Single Cell 3' Library, Gel Bead & Multiplex Kit and Chip Kit, version 2 (10× Genomics) according to the manufacturer's instructions. Sequencing was performed on an Illumina NextSeq6000 system (Genergy Biotechnology, Shanghai, China). Cell Ranger Suite version 2.1.1 was used to perform sample demultiplexing, barcode processing, and single-cell gene unique molecular index (UMI) counting. For quantification of single-cell gene expression and determination of the major cell types, single cells were filtered for downstream analysis by removing cells with fewer than 200 expressed genes, or over 10% UMIs derived from the mitochondrial genome. ECs were selected according to Cdh5 expression, for which gene expression matrices were normalized to total cellular and mitochondrial read counts. To reduce the dimensionality of the dataset, the resulting 1,500 variably expressed genes were summarized by principal component analysis (PCA) and the first 10 principal components were further summarized using t-distributed stochastic neighbor embedding (t-SNE) dimensionality reduction. Marker genes for each of the clusters were identified by overall correlation and PCA analysis; the Seurat FindMarkers function was then applied using a standard of > two-fold higher expression than the average expression in other clusters and *p* ≤ 0.05. Cell lineage trajectory analysis was performed by clustering the top 1,500 genes with the highest cluster specificity. These genes were used to compute pseudotime-ordering and cell trajectory using the Monocle 2 algorithm. The quality control data for scRNA-seq analysis are listed in supplementary Table S4.

### Quantitative reverse transcription polymerase chain reaction (qRT-PCR)

RNA was extracted using the TRIzol reagent (Invitrogen). cDNA was synthesized using a reverse transcription kit (Takara Biotechnology, Dalian, China). Real-time PCR was conducted using a SYBR Premix Ex Taq Kit (Takara) on an ABI PRISM7500 real-time PCR system (Life Technologies), with β-actin as an internal control. The primers are listed in supplementary Table S2.

### Western blotting

Cells were lysed in RIPA buffer (Beyotime) containing 10 mM phenylmethanesulfonyl fluoride (PMSF). Samples were separated by sodium dodecyl sulfate-polyacrylamide gel electrophoresis (SDS-PAGE), blotted onto polyvinylidene fluoride (PVDF) membranes, and probed with primary antibodies followed by HRP-conjugated secondary antibodies. β-ACTIN was used as a loading control. Membranes were developed using an enhanced chemo-luminescence (ECL) system (Clinx Science Instruments, Shanghai, China). Primary antibodies are listed in supplementary Table S3.

### Synthesis and characterization of nanoparticles

Polyethyleneimine (PEI)-polyethylene glycol (PEG) functionalized with the cyclo Arg-Gly-Asp-D-Phe-Lys (cRGD) (PEI-PEG-cRGD) nanoparticles were synthesized in Ruixi Biological Technology (Xi'an, China) according to the standard procedure 37. For morphological evaluation, PEI-PEG-cRGD nanoparticles were mixed with siMYC or NC and diluted to 10 μg/ml using diethylpyrocarbonate (DEPC) water. The nanoparticles were then coated with a gold-palladium sputter and the morphologies were determined by scanning electron microscopy (SEM) (Quattro S, Thermo Fisher, Rockford, Illinois, USA). To detect the size of nanoparticles, nanoparticles were prepared as described above and their size was measured using a Zetaview nanoparticle tracking analyzer (ParticleMetrix, Meerbusch, Germany) at 25 °C. The potential of nanoparticles was measured at the concentration of 4 mg/ml using a Zetaview nanoparticle tracking analyzer.

To investigate the encapsulation stability of PEI-PEG-cRGD nanoparticles, agarose gel retardation assay was performed. Briefly, nanoparticles were mixed with siMYC or NC and incubated at room temperature for 20 min. Samples were then subjected to electrophoresis with a 1% agarose gel in a Tris-acetate (TAE) running buffer at 140 V for 20 min. RNA was measured using a gel imaging system (Omega Fluor, Aplegen, CA, USA). RNA without nanoparticles was used as a negative control.

To evaluate the protection and preservation of RNA complexed with nanoparticles following systemic administration. siRNA-containing nanoparticles were incubated with 50% mouse serum at 37 °C for 0, 1, 3, 6, 12 or 24 h, respectively. The mixtures were then subjected to electrophoresis with a 1% agarose gel at 140 V for 20 min, and visualized using a gel imaging system.

To determine the transfection efficiency of nanoparticles, Cy3-labled siRNA was mixed with nanoparticles at room temperature for 10 min. The nanocomplexes were then added to HUVECs and incubated for 6 h. The medium was replaced with fresh ECM and continuously cultured for 12 h. Cy3 positive cells were visualized and captured under a fluorescent microscope. In order to investigate the *in vivo* transfection efficacy, Cy3-labeled siRNA was co-incubated with nanoparticles and administrated via tail vein injection into mice that had been inoculated with LLC for 14 days. Tumor tissues were collected at 3 h and 6 h post-injection for immunofluorescence staining, enabling analysis of the uptake of Cy3-labeled siRNA by CD31^+^ TECs.

To detect the cytotoxicity of nanoparticles *in vitro*, bEnd.3 cells were incubated with nanoparticles at different concentrations for 24 h. Cell counting kit - 8 (CCK8, MCE, Monmouth Junction, NJ, USA) (10 μl) was added to each well, and incubated at 37 °C for 4 h. Absorbance was determined at 450 nm using a microplate reader (Thermo Fisher). Cell viability was determined as the percentage of absorbance value compared with non-transfected cells. To evaluate the toxicity *in vivo*, blood samples were simultaneously harvested at the endpoint of sample collection, during which there were five administrations of nanoparticles. Serum was then isolated for subsequent blood chemistry analysis to evaluate the liver/kidney functions as measured by glutamic pyruvic transaminase (ALT), glutamic oxalacetic transaminase (AST), blood urea nitrogen (BUN) and creatinine (CR).

To investigate the hemolytic capacity of nanoparticles, fresh whole blood was harvested and anticoagulated using sodium heparin. Blood was then diluted 1:1 with saline. Nanoparticles were added into the diluted blood at different concentration and incubated at 37 °C for 1 h. Saline was used as a negative control, while deionized water was used as a positive control. Samples were centrifuged at 12000 rpm for 10 min. Supernatant was then collected and absorbance was determined at 456 nm using a microplate reader (Thermo Fisher).

For EC-targeted delivery of siMYC or miR-218 mimics *in vivo*, siMYC or miR-218 mimics (Ribobio) or NC was mixed with Cy5.5-labled PEI-PEG-cRGD nanoparticles (nucleic acid/ polymer = 1:3) and incubated for 10 min at room temperature. Nanoparticles with siRNA or mimics were then intravenously injected (4 mg/kg) every two days from day 7 after LLC tumor cell inoculation.

### Statistics

Statistical analysis was performed using Image-Pro Plus6.0, GraphPad Prism 8.0, SPSS 23 and GSEA2-2.2.3. All quantitative data are presented as means ± SD. Statistical significance was calculated using Student's *t*-tests for continuous variables between two groups. Comparison of continuous variables among more than two groups was performed by one-way ANOVA followed by Tukey's post hoc test for one independent variable. Nonparametric tests were used for non-normally distributed data. Probability values were two-tailed and *p* < 0.05 was considered statistically significant.

## Results

### Endothelial Notch signaling critically regulates tumor angiogenesis

**Figure 1:**
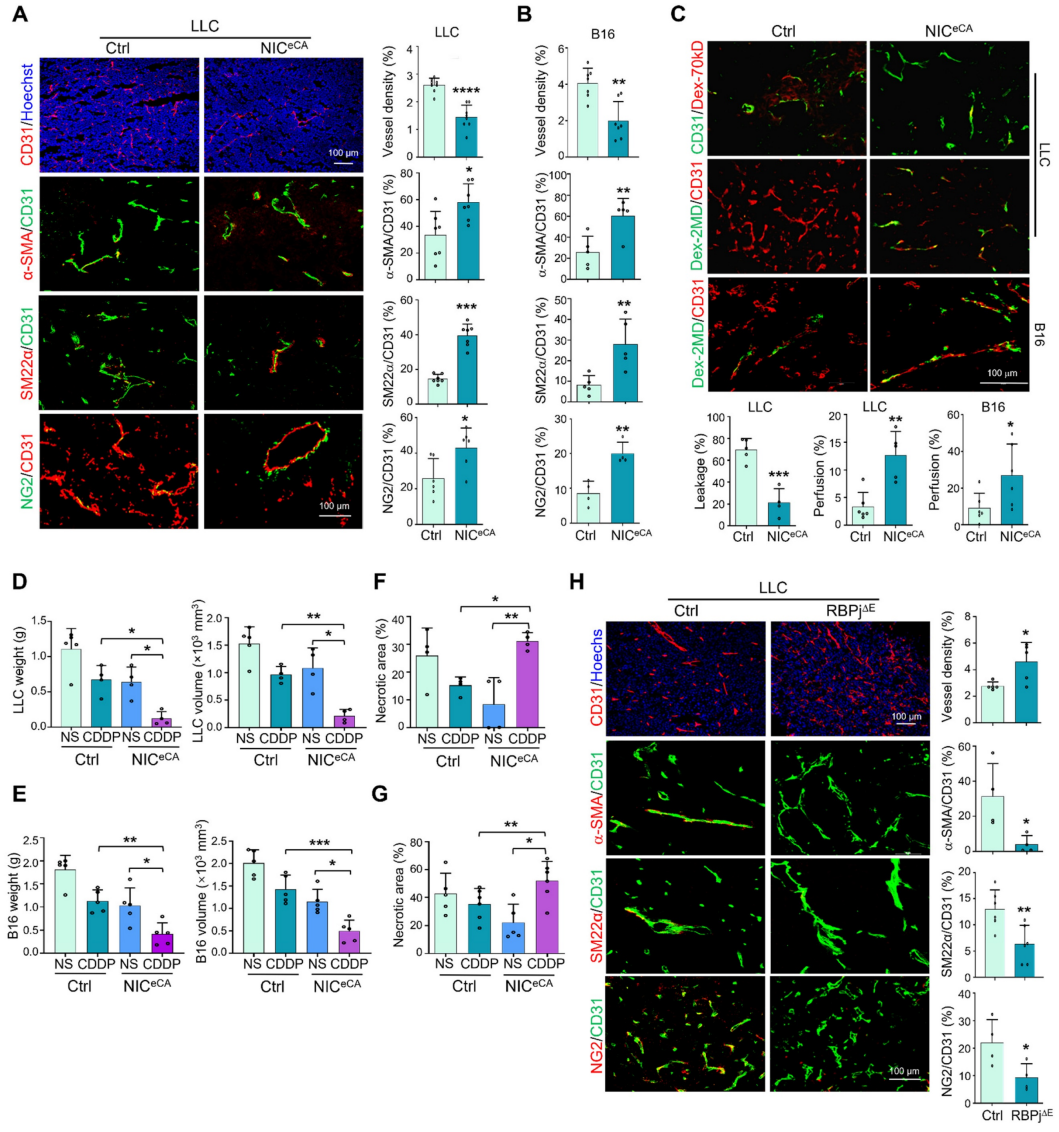
** Endothelial Notch activation represses tumor growth and promotes tumor vessel normalization.** (A) NIC^eCA^ and Ctrl mice were inoculated with LLC for 21 days. Tumor sections were stained with CD31, CD31 plus α-SMA, SM22α or NG2 immunofluorescence. Vessel density and ratio of α-SMA, SM22α or NG2 to CD31 were quantitatively compared between NIC^eCA^ and Ctrl groups (n = 7). (B) NIC^eCA^ and control mice were inoculated with B16 for 16 days. Tumor sections were stained with CD31, CD31 plus α-SMA, SM22α or NG2 immunofluorescence. Vessel density and ratio of α-SMA, SM22α or NG2 to CD31 were determined (n = 7). (C) LLC or B16 tumor-bearing mice were injected i.v with Dex-70kD or Dex-2MD. The percentages of leaky (CD31^+^Dex-70kD^+^) (n represents for at least 4) or perfused (CD31^+^Dex-2MD^+^) (n represents for at least 5) vessels were determined by immunofluorescence. (D and E) NIC^eCA^ and Ctrl mice were inoculated with LLC (D) or B16 (E). Mice were injected i.p every two days with NS or CDDP from 7 dpi. Tumor weight and volume was determined on 21 or 16 dpi (n represents at least 4). (F and G) Tumor sections from (D) and (E) were stained with H&E. Necrotic areas were quantitatively compared among the four groups (n represents at least 4). (H) RBPj^∆E^ and Ctrl mice were inoculated with LLC for 21 days. Tumor sections were stained with CD31, SM22α, α-SMA and NG2 immunofluorescence. Vessel density (n = 5) and pericyte/vSMC coverage (n = 4) were quantitatively determined. Bars = means ± SD. *, *p* < 0.05; **, *p* < 0.01; ***, *p* < 0.001; ****, *p* < 0.0001. Statistical tests: two-tailed Student's *t*-test for A - C, H; one-way ANOVA followed by Tukey's post hoc test for D - G.

To clarify the role of endothelial Notch signaling in tumor angiogenesis, we employed mice with tamoxifen-induced EC-specific NIC overexpression (NIC^eCA^, Cdh5-Cre^ERT^-ROSA-Stop^floxed^-*NIC*) or RBPj knockout (RBPj^∆E^, Cdh5-Cre^ERT^-*RBPj*^floxed^) to activate or block canonical Notch signaling in TECs, respectively (Figure S1A-D) 38. Endothelial Notch activation significantly repressed LLC and B16-F10 tumor growth as shown by decreased tumor weight and size accompanied by reduced tumor cell proliferation (Figure S1E-H). H&E staining and immunostaining showed that tumor necrosis and hypoxia were notably reduced under Notch activation (Figure S1G-H), consistent with normalized tumor vessels. Indeed, immunostaining showed that tumor vessel density decreased, while mural cell coverage increased significantly in NIC^eCA^ mice (Figure 1A-B, S1I). The vascular leakage and perfusion were evaluated with systemically administered Dextran-70kD or Dextran-2MD, respectively, and the results showed that tumor vessels in NIC^eCA^ mice exhibited decreased permeability and increased perfusion (Figure 1C). Next, NIC^eCA^ and Ctrl mice bearing LLC and B16 tumors were treated with cisplatin (CDDP) or saline (NS). The CDDP therapy displayed better efficacy with decreased tumor weight and volume in NIC^eCA^ mice (Figure 1D-E). Moreover, tumor necrosis significantly increased in the NIC^eCA^ plus CDDP group compared with the Ctrl plus CDDP group or the NIC^eCA^ plus NS group (Figure 1F-G, S1J-K). To the contrary, tumors in RBPj^∆E^ mice showed the opposite effects with increased tumor necrotic and hypoxic areas, enhanced vessel density and reduced mural cell coverage (Figure 1H, S1M), accompanied by decreased tumor weight and size (Figure S1L), due to destroyed tumor vessels as previously reported 23, 24. These data demonstrate that endothelial Notch activation promotes tumor vessel normalization and enhances chemotherapy efficacy.

### Notch activation targets a proliferating TEC subpopulation with high MYC level

We next isolated TECs using CD31 magnetic activated cell sorting (MACS) from the NIC^eCA^ and Ctrl mice, and performed RNA sequencing (RNA-seq) (Figure S2A). The result showed that Notch activation markedly altered TEC transcriptome involving multiple signaling pathways (Figure S2B-C). Morphogenesis-, tissue remodeling- and senescence-related genes are enriched in TECs with Notch activation, while cell cycle-related genes and MYC targets were downregulated (Figure S2D-E).

**Figure 2:**
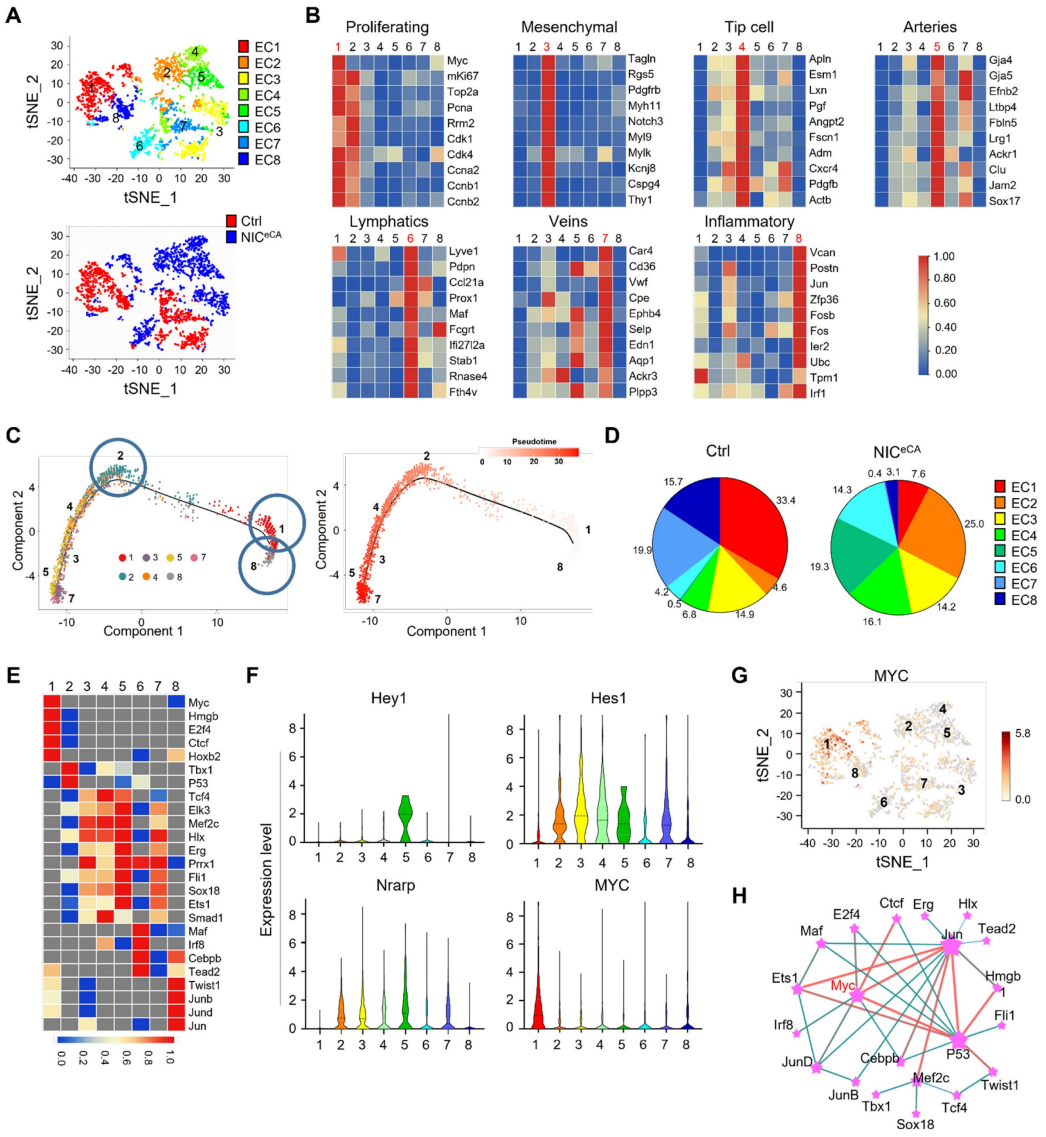
** scRNA-seq of TECs from NIC^eCA^ and control mice.** (A) t-SNE plots of Cdh5^+^ TECs. Each dot represents a single cell. The 8 TEC clusters are represented by different colors. Lower, TECs from NIC^eCA^ and Ctrl mice are represented by red and blue colors, respectively. (B) Heatmaps showing the expression of markers for proliferating ECs, mesenchymal ECs, tip cell, arteries, lymphatics, veins and activated ECs in cluster 1, 3, 4, 5, 6, 7 and 8, respectively. (C) Pseudotime analysis of the TEC cluster 1, 2, 3, 4, 5, 7 and 8. (D) Percentage of TECs from NIC^eCA^ (blue) and Ctrl (red) mice in each cluster. (E) Heatmap of *t*-values of the area under the curve (AUC) scores of expression regulation by transcription factors for different TEC clusters, as estimated using SCENIC. (F) Violin plots showing smoothened expression level of selected Notch downstream genes and MYC in TEC clusters. (G) MYC expression level in a single cell was shown in t-SNE dot. (H) Analysis of transcription factor interaction in (E) using the Omicsmart online tool.

To further elucidate how Notch activation regulates TECs, MACS-enriched CD31^+^ TECs were subjected to scRNA-seq. Totally 2,358 Cdh5^+^ TECs (1,095 from control and 1,263 from NIC^eCA^ mice) were recovered after excluding cell doublets and Cdh5^-^ cells, and Notch activation in the NIC^eCA^ group was confirmed by the up-regulated Nrarp and Hes1 (Figure S2F). TECs were clustered into 8 subpopulations according to their top expressed genes, as visualized by t-SNE plots (Figure 2A). These subpopulations were identified as proliferating ECs (cluster 1), mesenchymal-like ECs (cluster 3) 17, 39, tip cells (cluster 4), arterial ECs (cluster 5), lymphatic ECs (cluster 6), vein ECs (cluster 7), and inflammatory ECs (cluster 8). Cluster 2 expressed several general EC markers (CD31, Cdh5, CD34, Eng, and Emcn) but lacked expression of definitive EC subtype markers (Figure 2B, S2G). Among the identified clusters, cluster 1 demonstrated high proliferative capacity and elevated expression of MYC and proliferation-related genes, which appears to be a primary driver of tumor vascular abnormalities 8. Cluster 2 exhibited reduced MYC expression and decreased proliferative capacity relative to cluster 1. Cluster 4 was associated with angiogenesis initiation and played a pivotal role in vascular morphogenesis 40. Clusters 5 and 7 were predominantly composed of mature ECs, with cluster 5 being essential for establishing functional blood perfusion 31. Pseudotime trajectory analysis indicated these clusters likely originated from cluster 1 (the proliferating subpopulation), and cluster 1apperaed to have two different or functional directions, namely, the angiogenic direction (to clusters 2-4-3-5-7 with MYC-down) and the inflammatory direction (to cluster 8 with sustained MYC) (Figure 2C). Upon Notch activation, clusters 1, 7 and 8 were dramatically reduced, while clusters 2, 4 and 5 increased significantly (Figure 2D), which implies that Notch activation not only inhibits endothelial cell proliferation, reducing endothelial inflammation, but also promotes vascular morphogenesis and the formation of functional blood perfusion. Therefore, cluster 1, which accounted for about 30% TECs in the control, appears to be the critical target for Notch activation-induced tumor vessel normalization.

Single-cell regulatory network inference and clustering (SCENIC) analysis suggested that cluster 1 expresses high level of MYC regulon, consistent with its proliferating phenotype (Figure 2E). Further analysis showed that MYC mRNA is enriched in cluster 1, and in a reverse correlation with Notch downstream genes (Figure 2F-G) 17. Transcriptional network analysis revealed that MYC may functionally interact with JUN and p53 in TECs. These transcription factors are notably enriched in cluster 8 and 2, respectively, where JUN promotes inflammatory response while p53 suppresses cellular proliferation (Figure 2E and H) 41, 42. These results suggest that MYC expression and function in the proliferating cluster 1 could likely be the “Achilles' Heel” of TECs for tumor angiogenesis.

### Notch activation suppresses TEC activation by downregulating MYC expression

**Figure 3:**
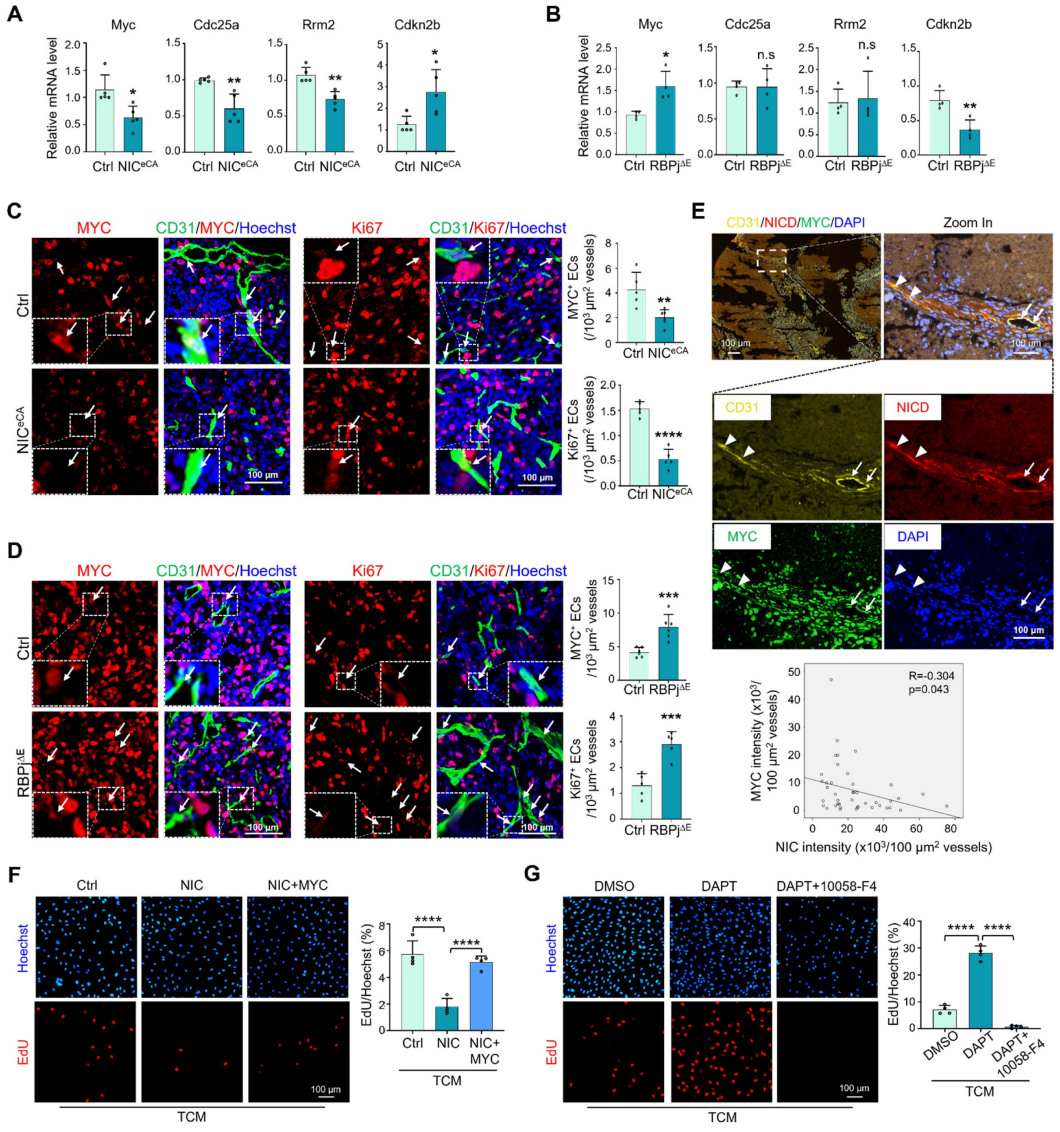
**Notch signal negatively regulates MYC expression in TECs.** (A) TECs were isolated from LLC tumors. The expression of MYC and its target genes between NIC^eCA^ and Ctrl mice was evaluated by qRT-PCR (n represents at least 5 for each gene). (B) TECs were isolated from LLC tumors. The expression of MYC and its target genes between RBPj^∆E^ and Ctrl mice was determined by qRT-PCR (n = 4). (C) LLC tumor sections from NIC^eCA^ and Ctrl mice were stained with CD31 plus MYC or Ki67 immunofluorescence. MYC^+^ and Ki67^+^ TECs was indicated by white arrows, and the number was quantitatively determined (n = 5). (D) LLC tumor sections from RBPj^∆E^ and Ctrl mice were stained with CD31 plus MYC or Ki67 immunofluorescence. MYC^+^ (n = 6) and Ki67^+^ (n = 5) TECs was indicated by white arrows, and the number was quantitatively evaluated. (E) Human lung cancer tissues were stained with multiplexed immunofluorescence. The correlation between NICD and MYC in CD31^+^ vessels was determined and compared (n = 45 fields from 12 human lung cancer tissues, white triangles represent CD31^+^NICD^+^MYC^-^ vessels, white arrows represent CD31^+^NICD^-^MYC^+^ vessels). (F) HUVECs were infected with adenovirus expressing NIC or control plus adenovirus expressing MYC or control, and cultured under TCM for 24 h. Cell proliferation was evaluated by EdU incorporation assay (n = 4). (G) HUVECs were treated with DAPT or DMSO plus 10058-F4 or DMSO, and cultured under TCM for 24 h. Cell proliferation was evaluated by EdU incorporation assay (n = 4). Bars = means ± SD. *, *p* < 0.05; **, *p* < 0.01; ***, *p* < 0.001; ****, *p* < 0.0001; n.s, not significant. Statistical tests: two-tailed Student's *t*-test for A - D; one-way ANOVA followed by Tukey's post hoc test for F and G.

In open transcriptome data 17, TECs exhibited higher proliferation and lower Notch signaling than normal endothelial cells (NECs) (Figure S3A), while MYC expression positively correlated with Ki67 and negatively correlated with Hey1 and normalization-related genes Cldn5 and Pdgfd (Figure S3B). TECs with higher Notch signaling displayed lower MYC activity and cell cycle progression (GSE51401) (Figure S3C). To experimentally validate the relationship between Notch and MYC in TECs, HUVECs were cultured in the presence of TCM, and transfected with NIC or treated with DAPT to activate or block Notch signaling, respectively. The results showed that Notch activation downregulated MYC expression and suppressed EC proliferation (Figure S3D-E, S3G-H), while Notch blockade showed opposite effects (Figure S3F, S3I-J). TECs were then isolated from NIC^eCA^, RBPj^∆E^, and control mice and subjected to qRT-PCR. The results showed that Notch activation decreased expression of MYC and its targets, and RBPj deficiency showed opposite effects (Figure 3A-B). At the protein level, immunofluorescence with CD31 plus MYC or Ki67 demonstrated that Notch activation significantly suppressed MYC expression and reduced proliferating (Ki67^+^) TEC (Figure 3C), while Notch blockade with RBPj deficiency upregulated MYC expression and increased TEC proliferation (Figure 3D). In a panel of human lung cancer biopsies, multiplexed immunofluorescence staining validated that Notch activation negatively correlated with MYC expression in TECs (Figure 3E). Moreover, MYC overexpression in HUVECs transfected with NIC rescued cell proliferation, and MYC blockade with an inhibitor (10058-F4) repressed cell proliferation induced by DAPT (Figure 3F-G, S3K) 43. These results suggest that Notch activation attenuates TEC proliferation via downregulating MYC, consistent with previously reports with angiogenic ECs 31, 32, 44.

### MYC inhibition corrects tumor vessel anomaly induced by RBPj deficiency

**Figure 4:**
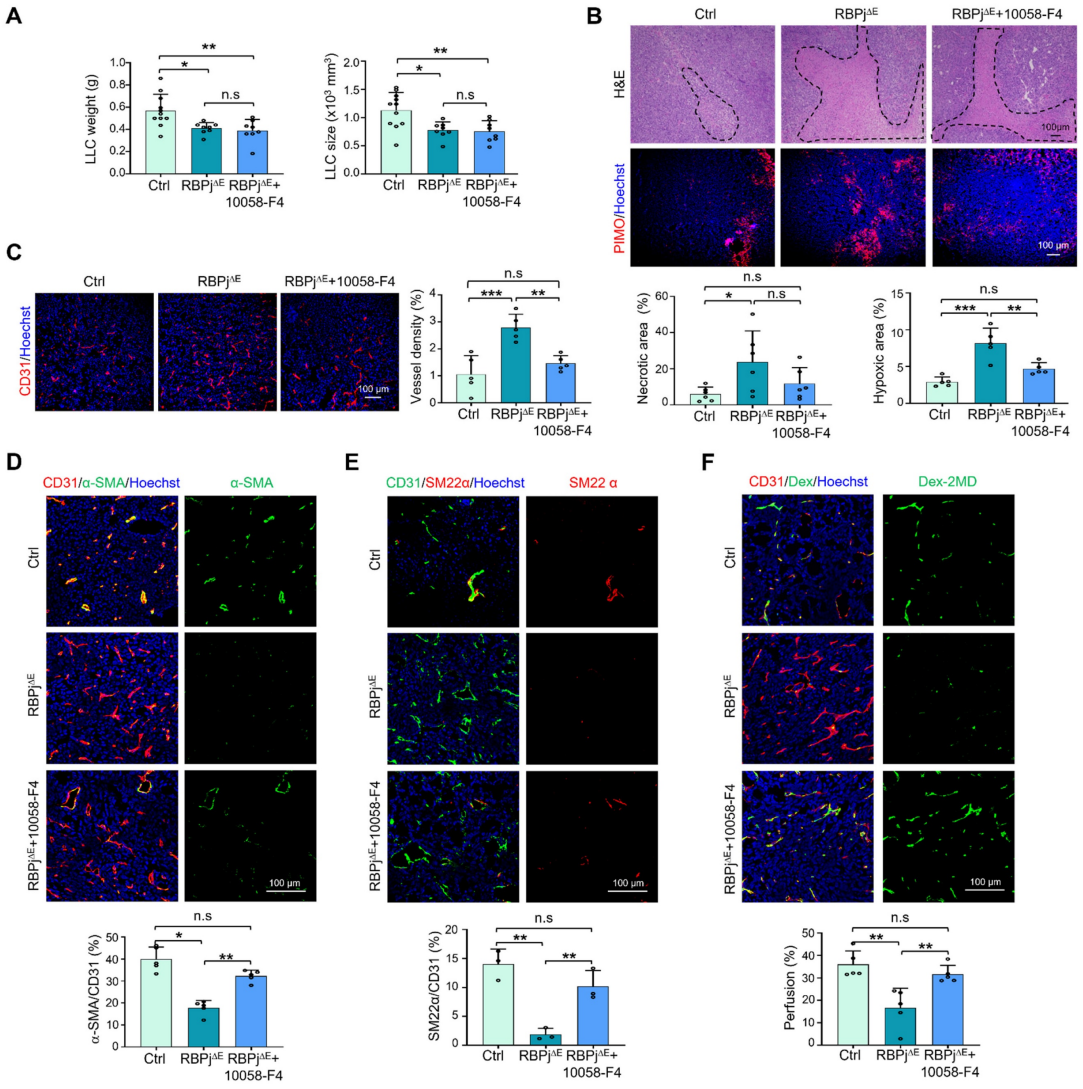
** MYC blockade with inhibitor normalizes tumor vessels under Notch blockade.** (A) RBPj^∆E^ and Ctrl mice were inoculated with LLC and treated with 10058-F4 or DMSO for 21 days. Tumor weight and size were measured and compared (n = 11 for Ctrl, n = 8 for RBPj^∆E^ and RBPj^∆E^ + 10058-F4). (B) Tumor sections were stained by H&E or PIMO. Tumor necrosis (n = 6) and hypoxia (n = 5) were quantified. (C - E) Tumor sections were stained with CD31, α-SMA, SM22α immunofluorescence. Vessel density (C) (n = 5) and pericyte/vSMC coverage (D and E) (n = 5 for α-SMA, n = 3 for SM22α) were determined. (F) LLC tumor-bearing mice were injected i.v with Dex-2MD. Percentage of perfused vessels were determined by immunofluorescence (n = 5). Bars = means ± SD. *, *p* < 0.05; **, *p* < 0.01; ***, *p* < 0.001; n.s, not significant. Statistical tests: one-way ANOVA followed by Tukey's post hoc test for A - F.

To evaluate the role of MYC in Notch-mediated regulation of TEC proliferation* in vivo*, mice bearing LLC were treated with the MYC inhibitor or vehicle. The results showed that 10058-F4 moderately normalized tumor vessels as shown by reduced tumor hypoxia, increased mural cell coverage and enhanced vessel perfusion, although this treatment minimally repressed tumor growth and tumor cell proliferation (Figure S4A-F). Then, we treated tumor-bearing RBPj^∆E^ or Ctrl mice with 10058-F4 or vehicle. The results revealed that, although the 10058-F4 treatment did not impact tumor growth, it markedly abolished the phenotype of enhanced tumor necrosis and hypoxia in RBPj knockout mice (Figure 4A-B, S4G). Tumor vessels, which were aggravated by RBPj deficiency, were normalized significantly by 10058-F4 as shown by reduced vessel density, increased mural coverage and vessel perfusion (Figure 4C-F). These results suggest that RBPj deficiency aggravates tumor vessel abnormality by MYC upregulation.

### EC-targeted siMYC nanoparticles promote tumor vessel normalization

To target MYC more precisely in TECs, we synthesized EC-targeted siRNA delivery nanoparticles, PEI-PEG-cRGD, which could specifically bind to TECs via integrin αVβ3 26, 37. The efficacy of siRNA targeting MYC was firstly tested *in vitro* (Figure S5A-B). Then, the properties of PEI-PEG-cRGD nanoparticles were evaluated. SEM revealed that PEI-PEG-cRGD nanoparticles were spherical, around 150 nm and 21.41 mV as validated by the ZETA analysis (Figure S5C-D). The gel retardation assay indicated that the nanoparticles could effectively encapsulate siMYC or negative control (NC) and protect them from degradation in serum for at least 12 h (Figure S5E-F). Moreover, nanoparticles could be efficiently taken in by bEnd.3 *in vitro* (Figure S5G), and no cell toxicity was detected except for at higher concentrations (Figure S5H). In addition, the nanoparticles showed no or low hemolytic activity as shown by the hemolytic analysis (Figure S5I-J) 45. Overall, these results suggest that the PEI-PEG-cRGD nanoparticles could efficiently encapsulate and transfer siRNA into ECs with acceptable safety 37.

**Figure 5:**
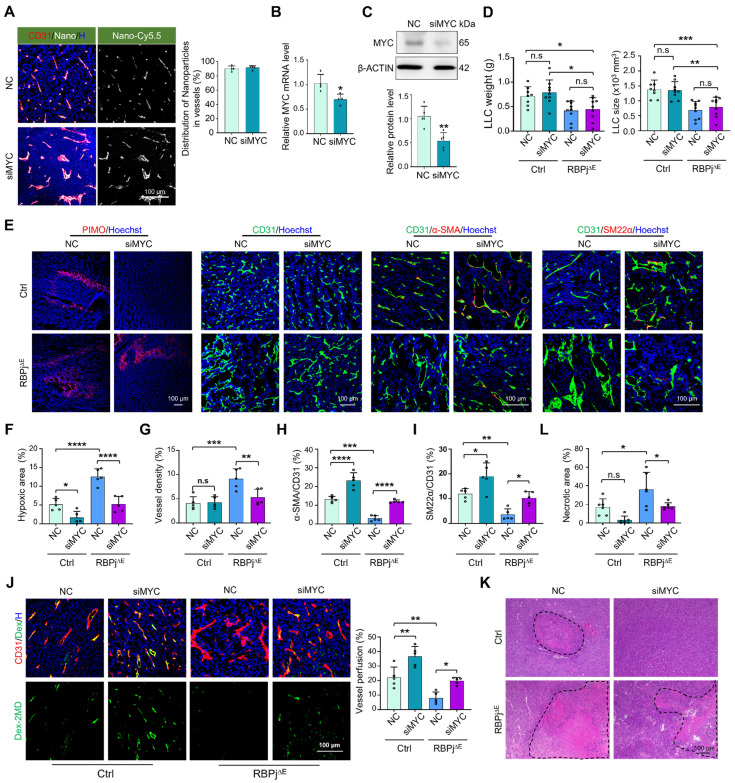
** EC-targeted MYC knockdown promotes tumor vessel normalization.** (A) Mice bearing LLC tumors was intravenously injected with Cy5.5-labeled siMYC or NC nanoparticles. The enrichment of Cy5.5^+^ nanocomplexes in CD31^+^ TECs was visualized via confocal microscope and subsequently quantified. (B and C) TECs were isolated using magnetic beads. MYC mRNA and protein levels were quantitatively determined by qRT-PCR (n = 4) (B) and western blotting (n = 5) (C), respectively. (D) RBPj^∆E^ and Ctrl mice bearing LLC tumors were treated with siMYC or NC. At day 21, tumor weight and size were measured and compared (n represents at least 8). (E - I) Tumor sections from (D) were stained with PIMO, CD31, α-SMA, SM22α immunofluorescence (E). Tumor hypoxia (F), vessel density (G) and pericyte/vSMC coverage (H and I) were quantitatively determined (n = 5). (J) LLC tumor-bearing mice were injected i.v with Dex-2MD. Vessel perfusion was determined by immunofluorescence (n = 5). (K and L) Tumor sections from (D) were stained with H&E. Tumor necrosis was measured and compared (n = 6). Bars = means ± SD. *, *p* < 0.05; **, *p* < 0.01; ***, *p* < 0.001; ****, *p* < 0.0001; n.s, not significant. Statistical tests: two-tailed Student's *t*-test for B and C; one-way ANOVA followed by Tukey's post hoc test for D, F-K.

Next, PEI-PEG-cRGD-encapsulated siMYC (EC-siMYC) or NC (EC-NC) was systemically administered to RBPj^∆E^ or Ctrl mice bearing LLC. Immunofluorescence staining of tumor tissues with CD31 showed that the nanocomplexes could specifically bind to TECs, with over 90% enrichment in CD31^+^ TECs (Figure 5A). Furthermore, siRNA delivery into TECs was achieved within 3 h (Figure S6A). EC-siMYC significantly downregulated MYC expression in TECs, as validated by qRT-PCR, western blotting and immunofluorescence (Figure 5B-C, S6B). EC-siMYC nanoparticles exhibited no significant effects on tumor weight and size in either RBPj^∆E^ or Ctrl mice (Figure 5D, S6C). However, EC-siMYC nanoparticles effectively normalized tumor vessels, as shown by reduced hypoxia, increased mural cell coverage and enhanced vessel perfusion in both RBPj^∆E^ and Ctrl mice, and decreased vessel density and necrosis in RBPj^∆E^ mice (Figure 5E-L, Figure S6D). Evaluation of tumor metastasis revealed that EC-siMYC reduced lung metastasis of LLC tumors, consistent with vascular normalization (Figure S6E) 46. Furthermore, the nanoparticles did not significantly alter the blood biochemical profile and the histology of heart, liver, kidney and lung in mice, confirming their safety (Figure S6F-G). Overall, these results demonstrate that EC-targeted siMYC nanoparticles represent a potential therapeutic strategy to normalize tumor vessels.

### miR-218 downstream to Notch signaling suppresses MYC expression in TECs and normalizes tumor vessels

**Figure 6:**
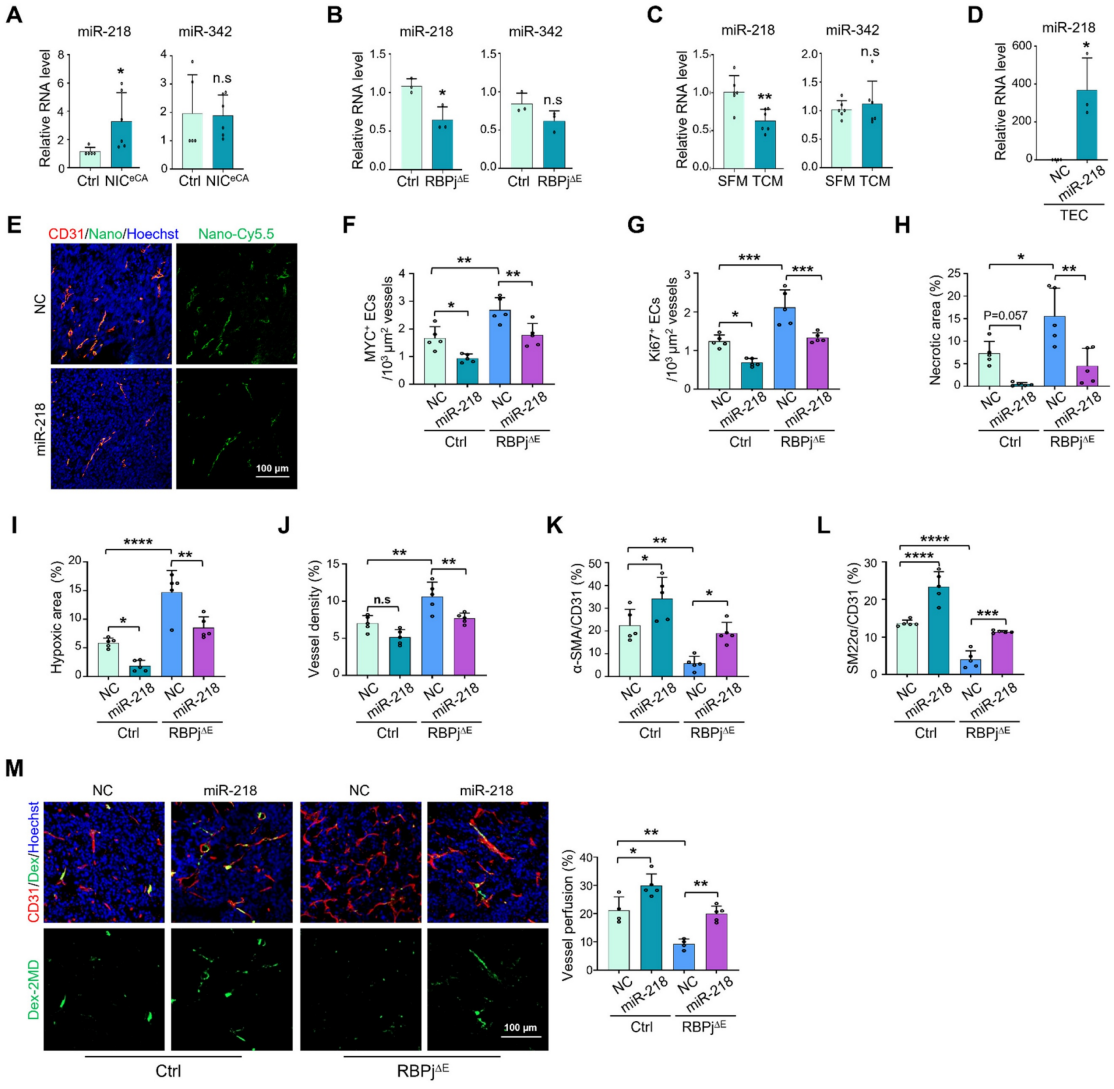
** miR-218 mediates the effects of Notch on suppressing MYC expression and normalizing tumor vessels.** (A) TECs were isolated from Ctrl and NIC^eCA^ mice bearing LLC tumors. The level of miR-218 and miR-342 was evaluated by qRT-PCR (n = 5). (B) TECs were isolated from Ctrl and RBPj^∆E^ mice bearing LLC tumors. The level of miR-218 and miR-342 was determined by qRT-PCR (n = 4). (C) HUVECs were treated with SFM or TCM from A549 tumor cells for 24 h. The level of miR-218 and miR-342 was evaluated by qRT-PCR (n = 6). (D) Mice bearing LLC tumors was injected with miR-218 or NC nanoparticles. TECs was isolated and the level of miR-218 was determined by qRT-PCR (n = 4 for NC, n = 3 for miR-218). (E) Mice bearing LLC tumors treated as in (D). Enrichment of Cy5.5^+^ nanocomplexes in vessels was captured under a confocal microscope. (F and G) Tumor sections from (D) were stained with CD31 plus MYC or Ki67. Number of MYC^+^ (F) and Ki67^+^ (G) TECs was quantitatively determined (n = 5). (H - L) Tumor sections from (E) were stained with H&E, GLUT1, CD31, α-SMA and SM22α immunofluorescence. Tumor necrosis (H), hypoxia (I), vessel density (J) and pericyte/vSMC coverage (K and L) were quantitatively evaluated (n = 5). (M) LLC tumor-bearing mice were injected i.v with Dex-2MD. Vessel perfusion was determined by immunofluorescence (n = 5). Bars = means ± SD. *, *p* < 0.05; **, *p* < 0.01; ***, *p* < 0.001; ****, *p* < 0.0001; n.s, not significant. Statistical tests: two-tailed Student's *t*-test for A - D; one-way ANOVA followed by Tukey's post hoc test for F - M.

We have previously shown that Notch signal inhibits MYC expression in ECs through upregulating miR-218 and miR-342-5p 30, 32. miRNAs hold potentials to normalize tumor vessels 47. We therefore accessed the role of these Notch-downstream miRNAs in TECs. The results showed that Notch activation upregulated miR-218 but not miR-342-5p expression in TECs, and Notch blockade exhibited the opposite effects (Figure 6A-B). Furthermore, miR-218 expression was significantly downregulated in HUVECs cultured with TCM (Figure 6C). Therefore, miR-218 might mediated the suppression of MYC by Notch signaling in TECs.

We then specifically delivered miR-218 or NC into TECs in RBPj^∆E^ or Ctrl mice using the PEI-PEG-cRGD-encapsulated miR-218 (EC-miR-218) or NC (EC-NC) nanoparticles (Figure 6D-E). The results showed that EC-targeted delivery of miR-218 significantly downregulated MYC expression, accompanied by repressed TEC proliferation (Figure 6F-G, S7C-D), although tumor growth was not obviously changed (Figure S7A-B). Furthermore, miR-218 notably reduced tumor necrosis and hypoxia (Figure 6H-I, S7E-F). miR-218 delivery markedly enhanced mural cell coverage and vessel perfusion in both RBPj^∆E^ and Ctrl mice (Figure 6K-M, S7H-I), accompanied by decreased vessel density in RBPj^∆E^ mice (Figure 6J, S7G). Similar to EC-siMYC, EC-miR-218 did not impact the overall functions of liver and kidney (Figure S7J). Altogether, these results show that miR-218 mediates the effects of Notch signaling on suppressing MYC in TECs and normalizing tumor vessels.

### MYC downregulation enhances the efficiency of chemo- and immuno-therapies

**Figure 7:**
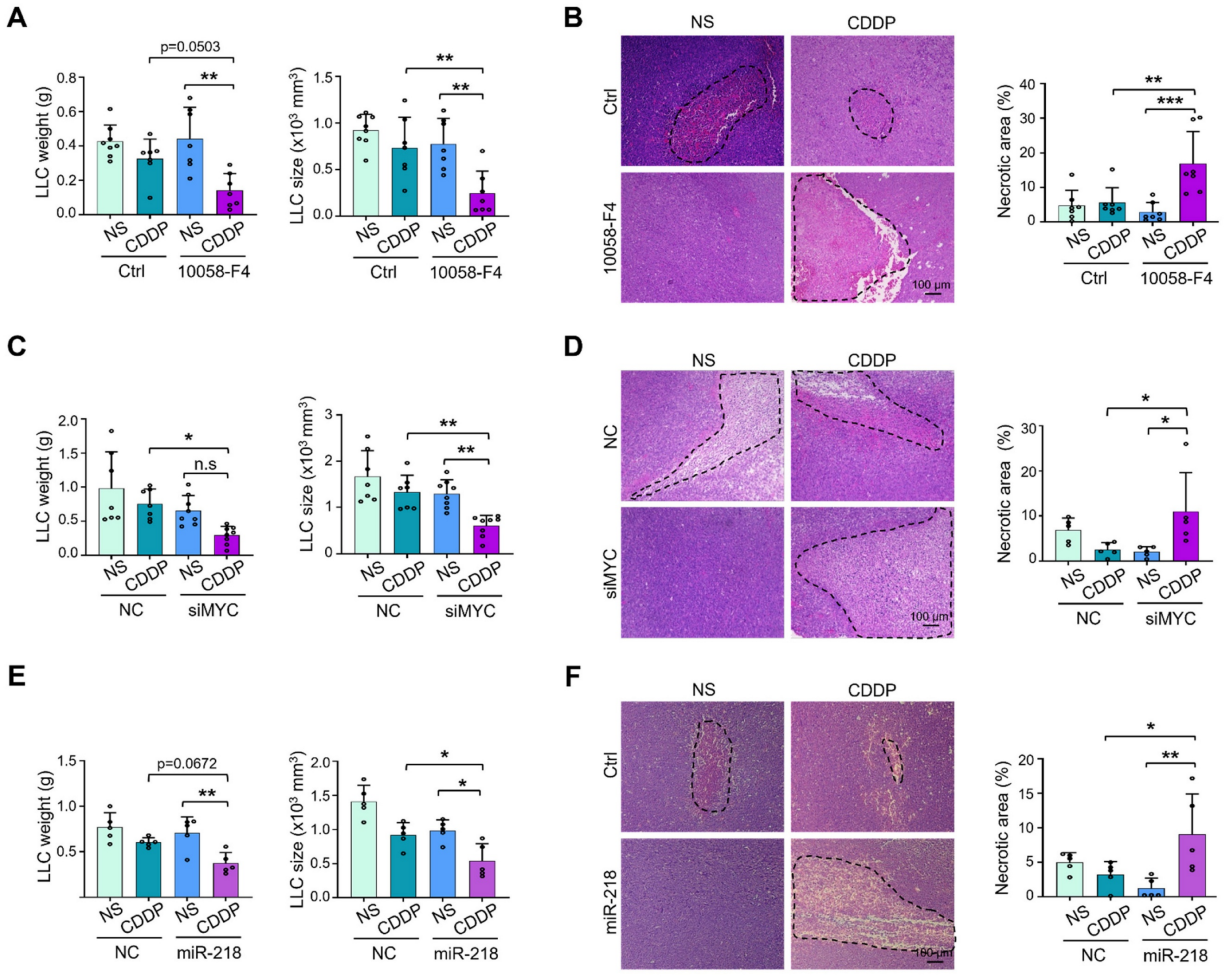
** Synergistic anti-tumor activities of combination therapy with MYC blockade and cisplatin.** (A) Mice bearing LLC tumors was treated with 10058-F4 or Ctrl plus NS or CDDP. Tumor weight and size were evaluated and compared (n represents at least 7). (B) Tumor sections from (A) were stained with H&E, and tumor necrosis was quantitatively determined (n = 7). (C) Mice bearing LLC tumors was treated with siMYC or NC nanoparticles plus NS or CDDP. Tumor weight and size were measured and compared (n = 7). (D) Tumor sections from (C) were stained with H&E. Tumor necrosis was measured and compared (n = 5). (E and F) Mice bearing LLC tumors were treated with miR-218 or NC nanoparticles plus NS or CDDP. Tumor weight and size (E) and tumor necrosis (F) were measured and compared (n = 5). Bars = means ± SD. *, *p* < 0.05; **, *p* < 0.01; n.s, not significant. Statistical tests: one-way ANOVA followed by Tukey's post hoc test for A - F.

To test whether TEC-specific MYC blockade enhances the sensitivity of chemotherapy, we treated LLC-bearing mice with 10058-F4, EC-siMYC or EC-miR-218 combined with CDDP or NS. The results showed that co-administration of 10058-F4 or EC-siMYC with CDDP exerted better efficacy on suppressing tumor growth, accompanied by increased tumor necrosis (Figure 7A-D, S8A-B). Similar phenotypes were observed upon coadministration of EC-miR-218 with CDDP (Figure 7E-F, S8C). These data suggest that MYC blockade may be a potential way to enhance the therapeutic effects of chemotherapy.

**Figure 8:**
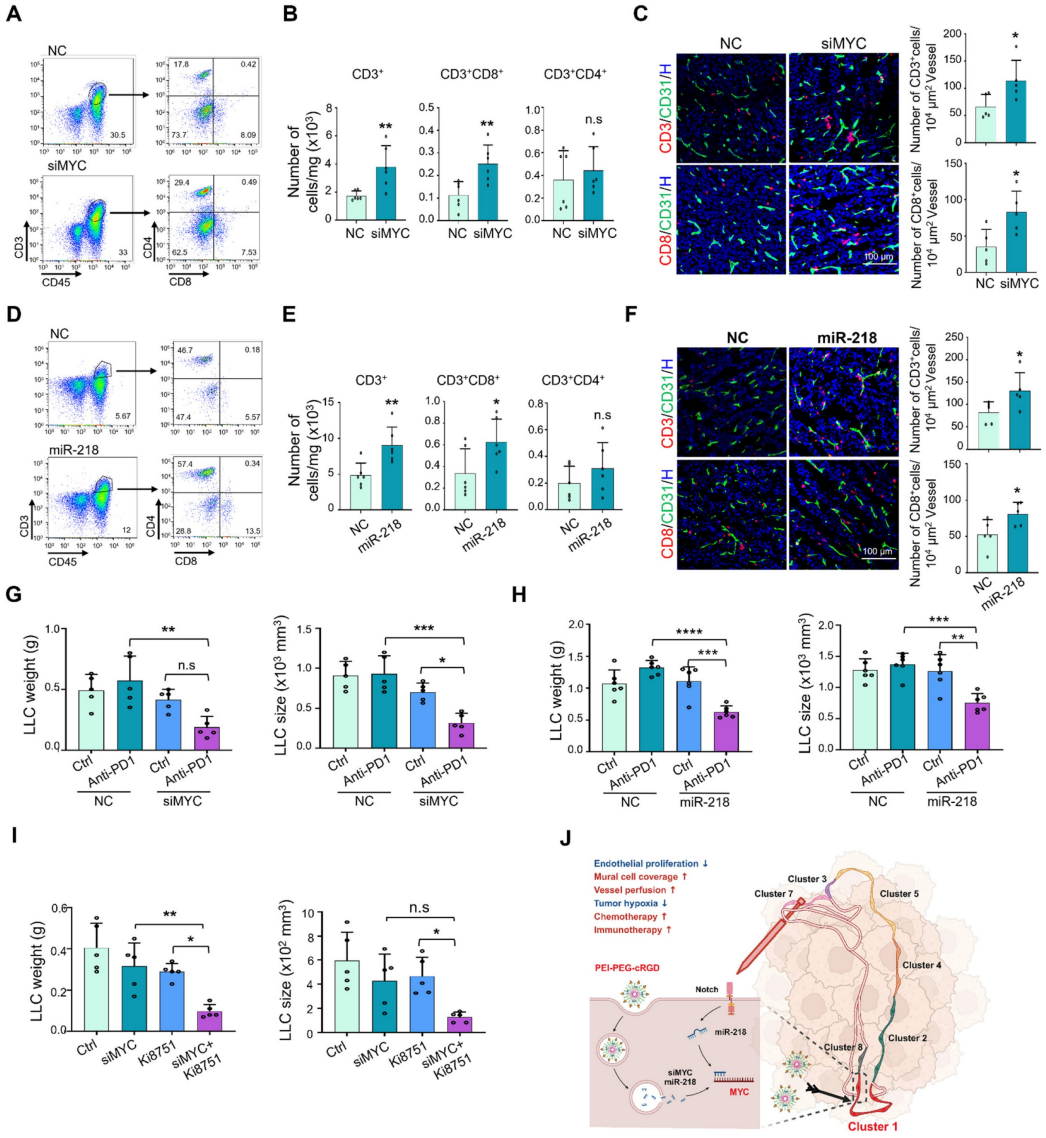
** EC targeted MYC blockade improves anti-PD1 immunotherapy.** (A and B) Mice bearing LLC tumors were treated with siMYC or NC nanoparticles for 21 days and subjected to flow cytometry. T cell staining pattern was shown in (A). Number of CD3^+^, CD3^+^CD8^+^ and CD3^+^CD4^+^ T cells in per mg tumor tissues was determined and compared (n = 6) (B). (C) Tumor sections from (A) were stained with CD31 plus CD3 or CD8 immunofluorescence. Number of CD3^+^ and CD8^+^ T cells around per 10^4^ μm^2^ vessels was counted and compared (n = 5). (D and E) Mice bearing LLC tumors were treated with miR-218 or NC nanoparticles for 21 days and subjected to flow cytometry. T cell staining pattern was shown in (D). Number of CD3^+^, CD3^+^CD8^+^ and CD3^+^CD4^+^ T cells per mg tumor tissues was evaluated (n = 6) (E). (F) Tumor sections from (A) were stained with CD31 plus CD3 or CD8 immunofluorescence. Number of CD3^+^ and CD8^+^ T cells around per 10^4^ μm^2^ vessels was counted and compared (n = 5). (G) Mice bearing LLC tumors were treated with EC-siMYC or EC-NC plus anti-PD1 antibody or saline (Ctrl). Tumor weight and size were measured and compared (n = 5). (H) Mice bearing LLC tumors were treated with EC-miR-218 or EC-NC plus anti-PD1 antibody or Ctrl. Tumor weight and size were measured and compared (n = 6). (I) Mice bearing LLC tumors were treated with EC-siMYC or EC-NC plus Ki8751 or DMSO. Tumor weight and size were measured and compared (n = 5). (J) Schematic diagram. Proliferating TEC (cluster 1) with high MYC expression is the Achilles' heel for chaos tumor vessels, and targeting endothelial MYC using EC-MYC or EC-miR218 might be an efficient strategy for AATs. Bars = means ± SD. *, *p* < 0.05; **, *p* < 0.01; ***, *p* < 0.001; ****, *p* < 0.0001; n.s, not significant. Statistical tests: two-tailed Student's *t*-test for B, C, E and F; one-way ANOVA followed by Tukey's post hoc test for G, H and I.

Next, we tested whether MYC blockade improves the immunotherapy with immune checkpoint inhibitors. We firstly assessed the effects of MYC blockade on tumor immune microenvironment using flow cytometry, and the results showed that EC-siMYC significantly promotes the infiltration of CD3^+^, CD3^+^CD8^+^ T cells and CD11b^+^F4/80^+^ macrophages (Figure 8A-B, S9A-C), whereas the CD3^+^CD4^+^ T cells, CD11b^+^Ly6G^+^ granulocytic myeloid-derived suppressor cells (MDSC) were not obviously affected (Figure S9A-C). The enhanced infiltration of CD3^+^ and CD8^+^ T cells was further validated by immunofluorescence staining (Figure 8C). Similar effects were also observed under EC-miR-218 delivery and endothelial Notch activation (Figure 8D-F, S9D-F), while Notch blockade exhibited the opposite effects (Figure S9G). Furthermore, we evaluated the effects of systemically administrated EC-siMYC or EC-miR-218 on the immunotherapy response by treating LLC-bearing mice with EC-siMYC or EC-miR-218 combined with anti-PD1 antibody. The results showed that both of EC-siMYC and EC-miR-218 enhanced LLC tumor response to anti-PD1 immunotherapy as shown by the decreased tumor growth in EC-siMYC or EC-miR-218 plus anti-PD1 group, while anti-PD1 immunotherapy alone provides no obvious therapeutic benefit in the LLC tumor model (Figure 8G-H, S9H-I). Endothelial Notch activation could also facilitate the efficacy of anti-PD1 treatment as shown in Figure S9J. We further explored the potential of MYC blockade in combining anti-VEGF therapy 36, and mice inoculated with LLC tumors were treated with EC-siMYC along with a VEGFR2 inhibitor (Ki8751). The results demonstrated that co-administration of EC-siMYC with Ki8751 exhibited superior efficacy in suppressing tumor growth (Figure 8I, S9K). These findings indicate that MYC blockade could serve as a promising strategy for enhancing the efficacy of anti-PD1 and anti-VEGF therapies.

## Discussion

The Notch signaling pathway plays pivotal roles in vessel morphogenesis and homeostasis by regulating EC proliferation, differentiation, and metabolism, and therefore has been repeatedly tested as a potential target of AATs 22, 48. Early attempts focused on blocking Notch signaling to disrupt tumor vasculature using Notch signal inhibitors. This strategy, although can successfully reduce tumor growth in mice, results in unacceptable side effect of disrupting vasculature and hemorrhage in normal tissues 3. More recently, it has been shown that in mice with constitutive endothelial Notch activation, tumor growth is also repressed with normalized tumor vasculature 26-29. However, Wieland et al demonstrated that endothelial Notch activation promotes metastasis by upregulating adherent molecules via a senescence mechanism 29. In fact, we also conducted preliminary experiments using the NIC^eCA^ mice, and no significantly increased lung metastasis was observed (data not shown). This discrepancy may be attributed to variations in tumor model manipulation or subtle differences in the background of the mouse strain employed 49. Nonetheless, these findings confirmed the value of Notch signaling as an AAT target, but distrusted its druggability. In the current study, we access this question by scRNA-seq analysis of TECs from NIC^eCA^ and Ctrl mice. Our data identified that Notch activation normalizes tumor vessels likely by repressing specifically a TEC subgroup with high proliferation. This TEC subset appears to give rise to angiogenic TEC subsets (clusters 2, 3, 4, 5 and 7) and the inflammatory TEC subset (cluster 8). Therefore, specifically targeting this TEC subset could be a promising strategy for AATs. Moreover, consistent with attenuated proliferating TEC subset, Notch activation reduced cell cycle-related gene expression by downregulating MYC. Previous studies have suggested that TECs exhibited higher MYC activity and cell cycle progression 13, which might be a potential target for tumor therapy 44. Recent reports by our group and others demonstrated that Notch signal acts as a negative regulator of MYC in ECs 30-32. In this study, our results further demonstrate that MYC was enriched in the proliferating TEC subset and was significantly downregulated under Notch activation in TECs. These findings position MYC as a promising target for AATs (Figure 8J).

MYC has been reported to directly or indirectly interact with other transcriptional factors, such as p53 and JUN. Prior studies have established that MYC and p53 participate in a reciprocal negative feedback loop, which plays a critical role in maintaining cellular homeostasis under both physiological and pathological conditions through multiple mechanisms 50, 51. Our recent published work has suggested that p53 activation, induced by nucleolar stress, promotes tumor vessel normalization 46. In the current study, we observed that elevated MYC expression in cluster 1 correlates with a low p53 level, whereas cluster 2 exhibits increased p53 expression, with a concomitant MYC downregulation. Direct interaction between MYC and p53 has not been documented at least in ECs, but a considerable amount of evidence has demonstrated noncoding RNAs, including miRNAs and lncRNAs, mediate MYC regulation on p53, and vice versa 52-54. Taken together, although our data support a model wherein MYC-down and concomitant p53-up likely facilitates a normal angiogenesis direction, the molecular mechanisms underlying crosstalk between MYC and p53 in this process remain to be clarified.

As for MYC and JUN, previous studies have documented correlated expression and functional interplay between MYC and JUN across various biological contexts 55, 56. In ECs, Strassheim et al. showed that c-Jun, MYC, and Foxo3 were coordinately upregulated in most pulmonary artery vasa vasorum (VV) endothelial cells (VVECs) in ATP-mediated VV angiogenesis, among which c-Jun was required for the expression of ATP-stimulated angiogenic genes, while MYC was repressive to anti-angiogenic genes 57. In our scRNA-seq data, the cluster 8 maintained an intermediate level of MYC expression but downregulated as compared with the cluster 1, accompanied by high-level of JUN. Given the established role of JUN in mediating inflammatory responses 42, our observation of heightened JUN expression in the inflammatory EC cluster is particularly noteworthy. Our data revealed that endothelial-specific MYC inhibition augments CD8^+^ T cell recruitment and potentiates anti-PD1 treatment efficacy. This phenotype could be a result of normalization of vessel morphology, which facilitates T cell infiltration and immunotherapy 58. However, while unresolved inflammation is a hallmark of the tumor microenvironment, emerging evidence indicates that endothelial inflammatory activation is critical for facilitating T cell infiltration and determining immunotherapy responsiveness 59. Further investigation is required to elucidate the role and molecular mechanisms governing the crosstalk between MYC and JUN in TECs, which may uncover novel targets for AATs.

As a master transcription factor controlling cell proliferation via multiple mechanisms in both normal and malignant cells, blocking MYC has been an important topic of developing small molecule anti-cancer drugs 43. In our hands, the MYC inhibitor could indeed normalize tumor vessels and exert synergetic effects on enhancing chemotherapy efficiency, consistent with previous studies 60. However, due to its lack of cell-type specificity, this approach may induce senescence in normal cells, elicit multiple adverse effects, and potentially activate adaptive signaling pathways linked to therapeutic resistance 61. To overcome these limitations, we developed an EC-targeted nanoparticle system (PEI-PEG-cRGD) that specifically binds to TECs through integrin αVβ3 interaction, enabling efficient nucleic acid delivery with tolerable toxicity 37, 62, 63. As cargos, our results showed that the delivery of the MYC siRNA notably downregulated the MYC expression in TECs, accompanied by tumor vessel normalization in both RBPj^∆E^ and Ctrl mice, confirming that MYC is an authentic downstream target for Notch activation to repress TEC proliferation. Meanwhile, unlike the strong side effects caused by Notch signal disruption 6, EC-siMYC has no obvious side effects on normal tissues. Therefore, MYC blockade in TECs by nanoparticle-mediated siRNA delivery is an alternative way to normalize tumor vessels (Figure 8J). Our findings demonstrate that MYC inhibition in TECs significantly enhances the coverage of mural cell, potentially due to the upregulation of PDGFD, a molecule involved in mural cell recruitment 64, 65. However, further investigation is required to elucidate the precise mechanism by which endothelial MYC inhibition regulates mural cell recruitment. This nano-based strategy may have several advantages. Firstly, despite the availability of diverse AATs, such as antibodies, small molecule inhibitors, and adeno-associated viruses (AAVs), nanoparticle-based delivery systems offer distinct advantages in terms of selectivity and efficacy 66. By modulating parameters such as size, morphology, and surface functionalization, these nanoplatforms can effectively bypass biological barriers, enhance tissue penetration, and enable precise, controlled drug delivery 66. Secondly, it targets a higher fraction of TECs than those cell surface receptor inhibitors. Our and others' scRNA-seq data 17 showed that around 30% of TECs are enriched in MYC and this proliferating TEC subset appears to give rise to most, if not all, other TEC subsets, functioning as the Achilles' heel of the tumor vascular system. In contrast, inhibitors of VEGFR2, the representative angiogenic cell surface receptor in TECs, target less than 10% of TECs 17, 18. Thirdly, different cell surface receptors, such as VEGFR2, MET, TIE2, WNT, and chemokine receptors, which are all enriched in tumor microenvironment, converge their signal transduction pathways on MYC to promote TEC proliferation 31, 44. Targeting MYC will likely recapitulate their effects on TECs and provide a more efficient strategy for AATs. Indeed, our results showed that combination of MYC inhibition with anti-VEGF inhibitors in TECs exhibited enhanced efficacy in suppressing tumor growth. However, the relationship between VEGF-VEGFR2 signaling pathway and MYC expression appears to be relatively complex in ECs, and increased VEGF signaling could suppress EC proliferation to induce arterialization through downregulating MYC expression. Fourthly, MYC is essential not only for the majority of cancer cells but also for tumor environmental cells, including anti-tumor immune cells like T and NK lymphocytes 67, 68. TEC-targeting anti-MYC nanoparticles can prevent inadvertent damage to immune cells. In summary, nanoparticle-based delivery systems hold significant promise for advancing precision medicine. However, their clinical translation remains hindered by key challenges, such as suboptimal biodistribution, rapid immune clearance, and unintended immunogenicity 69. Addressing these limitations may require further research into advanced surface functionalization strategies or the development of novel nanoparticles 69.

The mechanism underlying Notch activation-induced MYC repression in TECs has not been completely understood. Our recent data have shown that Notch may repress MYC expression at different levels by upregulating a group of miRNAs, notably, miR-218-5p and miR-342-5p 30, 32. However, in TECs, Notch signaling seems to upregulate miR-218 but has no obvious effects on miR-342. We therefore also tested the effect of TEC-specific nanoparticle-mediated delivery of miR-218. The results showed that similar as the MYC siRNA, delivery of miR-218 also suppresses MYC expression in TECs and normalizes tumor vessels. This finding lends further support for the Notch-miR-218-MYC pathway in regulating EC proliferation, and provides another tool for TEC-specific nanoparticle-mediated MYC suppression. Notably, while miR-218 mediates Notch-induced MYC suppression in TECs, it fails to reduce tumor growth. This could be attributed to the efficiency of EC-targeted delivery and/or the efficiency of miR-218-mediated MYC inhibition. Given the complexity of MYC's upstream regulatory work 70, it is likely that there exist additional, miR-218 independent mechanisms of MYC regulation in promoting tumor growth. Further mechanistic studies are required to elucidate the precise role of miR-218 in TECs. Previous study also indicated that MYC is a direct target gene of Notch 71, implying the involvement of other potential mechanisms such as transcriptional repression. Therefore, comprehensive mechanistic studies, including luciferase reporter assays, chromatin immunoprecipitation (ChIP), ribosome profiling (Ribo-seq), and high-resolution mass spectrometry (MS), are warranted to delineate the precise Notch-MYC regulatory axis across transcriptional, translational, and post-translational levels in ECs. Additionally, the relationship between Notch and miR-342-5p in TECs may be more intricate. Our recent study suggests that miR-342-5p exerts a negative regulatory effect on Notch signaling in vSMCs, thereby plays an antagonistic role 72. Considering the presence of numerous potential downstream target genes of miRNA, which are influenced by various intracellular factors, further exploration is required to elucidate the relationship between miR-342-5p and Notch in TECs. Moreover, whether targeted delivery of miR-342-5p can also exhibit similar functionality as miR-218 remains to be clarified.

AATs have been reported to improve the efficiency of chemotherapy and immunotherapy through enhancing infiltration of drugs or immune cells 73. Han et al previously reported that MYC inhibitors could suppress tumor growth and enhance immunotherapy 74. In this study, both MYC siRNA and EC-miR-218 significantly promote infiltration of CD3^+^ and CD8^+^ T cells, and combination of TEC-specific MYC blockade and immunotherapy does exhibit better inhibition effects than anti-PD1 antibody treatment alone. ECs have long been known to participate in immune response by regulating immune cell trafficking, activation and function 75. Recently, Qi et al have reported that endothelial MYC knockout promotes pro-inflammatory response and increases leukocyte infiltration 76, suggesting that MYC blockade may enhance immune cell infiltration through multiple mechanisms, such as overcoming endothelial cell anergy 58, 77, in addition to improving vascular perfusion. Therefore, further exploration is needed to understand the mechanism by which MYC blockade improves the immunomodulatory functions of ECs 58. Additionally, given MYC's well established role in driving tumor progression, and the demonstrated ability of MYC inhibition to suppress tumor cell proliferation and impede tumor growth via specific inhibitors 43, 74, it would be highly valuable to investigate whether dual targeting of MYC in both TECs and tumors cells could yield synergistic and sustained anti-tumor effects.

Challenges and limitations: Given the well-documented heterogeneity of TECs across varying TME 78, comprehensive clinical validation is required but not limited to facilitate the translation of these preclinical findings to patients: systematically quantify MYC expression levels in TECs from diverse human cancer types; establish robust correlations between MYC expression and endothelial proliferation, inflammatory response signatures, anti-angiogenic treatment outcome, and immunotherapy response profiles. In the current study, our reliance on LLC and B16-F10 models, while experimentally tractable, poorly recapitulates human tumor complexity. Therefore, further validation using orthotopic tumor models, spontaneous tumor models and humanized tumor models is warranted in subsequent research 79.

In summary, our study has elucidated the role of endothelial Notch-MYC pathway in TECs at the single cell level, and established TEC-targeted nanoparticles to normalize tumor vasculature by suppressing MYC. Targeting endothelial MYC using nanoparticles bearing siRNA or miRNA is an efficient strategy for tumor anti-angiogenic therapy to enhance the efficacy of chemo- and immune checkpoint inhibitor-based immuno-therapies.

## Supplementary Material

Supplementary figures and tables.

## Abbreviations

AATs: anti-angiogenic therapies
VEGFR2: vascular endothelial growth factor receptor 2
ECs: endothelial cells
TECs: tumor ECs
TME: tumor microenvironment
scRNA-seq: single-cell RNA sequencing
cRGD: cyclo Arg-Gly-Asp-D-Phe-Lys peptide
RBPj: recombination signal binding protein Jkappa
SPF: specific pathogen-free
OCT: optimal cutting temperature
PFA: paraformaldehyde
BSA: bovine serum albumin
TSA: tyramide signal amplification
ATCC: American type culture collection
DMEM: dulbecco's modified eagle's medium
FCS: fetal calf serum
HUVECs: human umbilical endothelial cells
FBS: fetal bovine serum
ECGS: endothelial cell growth supplements
MOI: multiplicity of infection
DMSO: dimethyl sulfoxide
TCM: tumor cell derived conditioned medium
SFM: serum-free medium
EdU: 5-Ethynyl-2'-deoxyuridine
rRNA: ribosomal RNA
UMI: unique molecular index
PCA: principal component analysis
qRT-PCR: quantitative reverse transcription polymerase chain reaction
t-SNE: t-distributed stochastic neighbor embedding
PMSF: phenylmethanesulfonyl fluoride
SDS-PAGE: sodium dodecyl sulfate-polyacrylamide gel electrophoresis
PVDF: polyvinylidene fluoride
ECL: enhanced chemo-luminescence
PEI: polyethyleneimine
PEG: polyethylene glycol
DEPC: dithylpyrocarbonate
TAE: tris-acetate
CCK8: cell counting kit-8
ALT: glutamic pyruvic transaminase
AST: glutamic oxalacetic transaminase
BUN: blood urea nitrogen
CR: creatinine
LLC: lewis lung carcinoma
CDDP: cisplatin
NS: saline
MACS: magnetic activated cell sorting
SCENIC: single-cell regulatory network inference and clustering
NECs: normal endothelial cells
SEM: scanning electron microscopy
NC: negative control
EC-siMYC: PEI-PEG-cRGD-encapsulated siMYC
EC-NC: PEI-PEG-cRGD-encapsulated NC
MDSC: granulocytic myeloid-derived suppressor cells
7-AAD: 7-aminoactinomycin D
ChIP: chromatin immunoprecipitation
Ribo-seq: ribosome profiling
MS: mass spectrometry
AAVs: adeno-associated viruses
