# Supplementary material for: Targeting endothelial MYC using siRNA or miR-218 nanoparticles sensitizes chemo- and immuno-therapies by recapitulating the Notch activation-induced tumor vessel normalization
Source: Theranostics. 2025 Apr 13;15(11):5381–401. doi: 10.7150/thno.112023 (PMC12036866; doi:10.7150/thno.112023)
Supplement: Supplementary file 1 — Supplementary figures and tables. [file thnov15p5381s1.pdf]

Supplementary information

Supplementary Table S1. Information of lung cancer patients involved in this study.

No.	Sex	Age (years)	Tumor size (cm^3)	Tumor grade
1	M	54	2×1.5×1.5	II
2	M	65	6×6×6	III
3	M	48	2.5×2×2	II
4	F	60	4.5×3×2	?
5	F	75	1.8×1.5×1.5	?
6	M	61	4×2×1.5	II
7	M	61	2.5×2×2	II
8	M	39	5×4×4	II-III
9	M	69	4×4×3.5	III
10	M	77	4×3.5×3	III
11	F	66	3×2×1.5	II
12	M	68	6×4.5×3	II-III

*M, male; F, female; ?, information missing.

Supplementary Table S2. Primers used in the study.

**Primer name**	**Sequence (5’-3’)**
Mouse Hey1 forward (qRT-PCR)	CATGAAGAGAGCTCACCCAGA
Mouse Hey1 reverse (qRT-PCR)	CGCCGAACTCAAGTTTCC
Mouse β-Actin forward (qRT-PCR)	GGCTGTATTCCCCTCCATCG
Mouse β-Actin reverse (qRT-PCR)	CCAGTTGGTAACAATGCCATGT
Mouse Rrm2 forward (qRT-PCR)	TGCGAGGAGAATCTTCCAGGAC
Mouse Rrm2 reverse (qRT-PCR)	CGATGGGAAAGACAACGAAGCG
Mouse Cdc25a forward(qRT-PCR)	CCTACTGATGGCAAGCGTGTCA
Mouse Cdc25a reverse(qRT-PCR)	CTCATTGCCGAGCCTATCTCTC
Mouse Cdkn2b forward(qRT-PCR)	ATCCCAACGCCCTGAACCGCT
Mouse Cdkn2b reverse(qRT-PCR)	AGTTGGGTTCTGCTCCGTGGAG
Mouse Myc forward(qRT-PCR)	TCGCTGCTGTCCTCCGAGTCC
Mouse Myc reverse(qRT-PCR)	GGTTTGCCTCTTCTCCACAGAC
Human Hey1 forward (qRT-PCR)	TGTCTGAGCTGAGAAGGCTGGT
Human Hey1 reverse (qRT-PCR)	TTCAGGTGATCCACGGTCATCTG
Human β-Actin forward (qRT-PCR)	TGGCACCCAGCACAATGAA
Human β-Actin reverse (qRT-PCR)	CTAAGTCATAGTCCGCCTAGAAGCA
miR-218-5p forward (qRT-PCR)	GGCTTGTGCTTGATCTAACCATGT
miR-218-5p reverse (qRT-PCR)	NA
miR-342-5p forward (qRT-PCR)	CGGAGGGGTGCTATCTGTGATTGAG
miR-342-5p reverse (qRT-PCR)	NA
U6 forward (qRT-PCR)	GGATGACACGCAAATTCGTGAAGC
U6 reverse (qRT-PCR)	NA
CreN1 (Genotype)	CCGGTCGATGCAACGAGTGATGAGG
CreN2 (Genotype)	GCCTCCAGCTTGCATGATCTCCGG
N1 Common (Genotype)	AAAGTCGCTCTGAGTTGTTAT
N1 Wild type (Genotype)	TAAGCCTGCCCAGAAGACTC
N1 Mutant (Genotype)	GAAAGACCGCGAAGAGTTTG

R3 (Genotype)	GTTCTTAACCTGTTGGTCGGAACC
R4 (Genotype)	GCTTGAGGCTTGATGTTCTGTATTGC
PGKD (Genotype)	ACCGGTGGATGTGGAATGTGT
siRNA1# sense	GACGAGACCUUCAUCAAGATT
siRNA1# antisense	UCUUGAUGAAGGUCUCGUCTT
siRNA2# sense	GAGGAUAUCUGGAAGAAAUTT
siRNA2# antisense	AUUUCUUCCAGAUAUCCUCTT
siRNA3# sense	GAGGAUAUCUGGAAGAAAUTT
siRNA3# antisense	AUUUCUUCCAGAUAUCCUCTT

Supplementary Table S3. Antibodies used in the study.

Product name	Company	Product code
Anti-CD31	BD Pharmingen	550274
Anti-SM22α	Abcam	Ab14106
Anti-α-SMA	Abcam	Ab124964
Anti-NG2	Milipore	AB5320
Anti-Ki67	Milipore	AB9260
Anti-MYC	CST	13987
Anti-β-ACTIN	Proteintech	66009-1-Ig
Anti-NICD	CST	4147
BV510-anti-mouse CD45	Biolegend	157219
APC-anti-mouse CD3	Biolegend	100236
FITC-anti-mouse CD8a	Biolegend	100706
PE-anti-mouse CD4	Biolegend	100408
FITC-anti-mouse/human CD11b	Biolegend	101206
APC/Cy7-anti-mouse Ly6G	Biolegend	127624
APC-anti-mouse F4/80	Biolegend	157306
Alexa Fluor® 488-anti-rat IgG	Invitrogen	A-21208
Alexa Fluor®594-anti-rat IgG	Invitrogen	A-11007
Alexa Fluor®594-anti-rabbit IgG	Jackson	711-586-152
Alexa Fluor®488-anti-rabbit IgG	Jackson	711-545-152
iF440-Tyramide	Servicebio	G1250
iF488-Tyramide	Servicebio	G1231
iF546-Tyramide	Servicebio	G1251
iF594-Tyramide	Servicebio	G1233
iF700-Tyramide	Servicebio	G1252
HRP-anti-rabbit IgG	Proteintech	SA00001-9
HRP-anti-mouse IgG	Proteintech	SA00001-8

Supplementary Table S4. Quality control data for single-cell sequence analysis

Group	Ctl	NIC^{eCA}
Estimated number of cells	2302	4455
Median genes per cell	2531	2060
Mean reads per cell	147159	60465
Median UMI counts per cell	7164	5679
Fraction reads in cells	62.2%	66.3%
Sequencing Saturation	83.9%	69.6%
Q30 bases in barcode	97.2%	97.6%
Q30 bases in RNA read	94%	95.1%
Q30 bases in sample index	96.5%	95.8%
Q30 bases in UMI	95.9%	96.5%

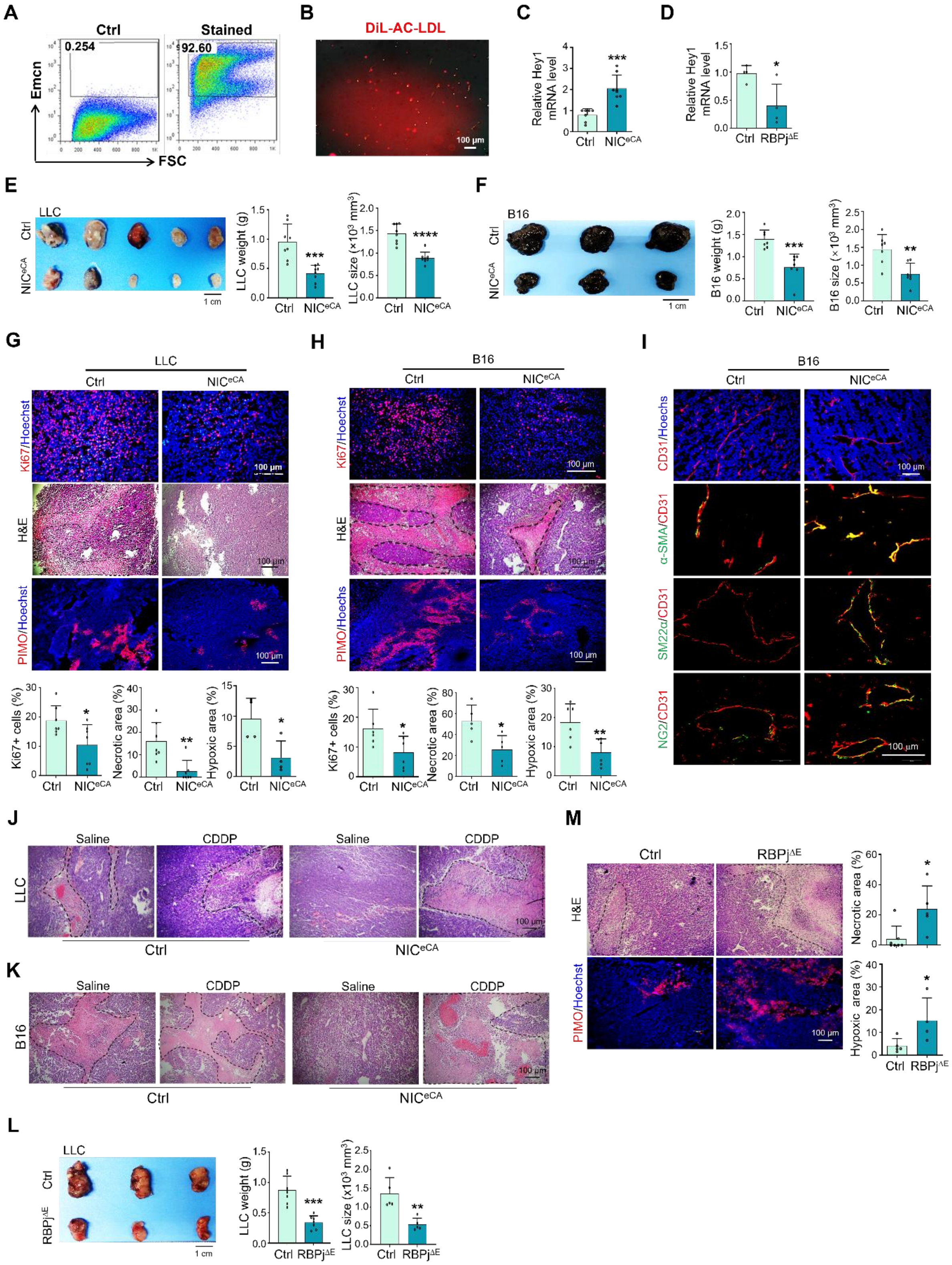

Figure S1. Endothelial Notch activation represses tumor growth and normalizes tumor vessel. (A and B) TECs was isolated using anti-CD31-coated beads. Cells were analyzed by flow cytometry (A) and Dil-Ac-LDL uptake assay (B). (C) NIC^{eCA} and control mice were inoculated with LLC. TECs were isolated at day 21, and Hey1 mRNA level was determined

by RT-qPCR (n = 8). (D) $RBPj^{\Delta E}$ and Ctrl mice were inoculated with LLC. TECs were isolated at day 21, and Hey1 mRNA level was determined by qRT-PCR (n = 4). (E) NIC^{eCA} and Ctrl mice were inoculated with LLC. Tumors were harvested and photographed, and tumor weight and size were determined at day 21 (n = 8). (F) NIC^{eCA} and control mice were inoculated with B16. Tumors were harvested and photographed, and tumor weight and size were determined at day 16 (n = 7). (G) LLC tumor sections from (E) were stained with Ki67 immunofluorescence, H&E and PIMO immunofluorescence. $Ki67^+$ cells (n = 7), necrotic (n = 7) and hypoxic (n = 4) areas were quantitatively compared. (H) B16 tumor sections from (F) were stained with Ki67 immunofluorescence, H&E and PIMO immunofluorescence. $Ki67^+$ cells (n = 6), necrotic (n = 5) and hypoxic (n = 6) areas were quantitatively compared. (I) B16 tumor sections from (F) were stained with CD31 plus α-SMA, SM22α or NG2 immunofluorescence. The ratio of α-SMA, SM22α or NG2 to CD31 was determined (n = 7). (J and K) NIC^{eCA} and Ctrl mice were inoculated with LLC (J) or B16 (K) and treated with saline (NS) or cisplatin (CDDP) from day 7. Representative images showed the tumor necrosis in different groups. (L) $RBPj^{\Delta E}$ and Ctrl mice were inoculated with LLC for 21 days. Tumor weight (n = 7) and volume (n = 5) were determined. (M) Tumor sections from (L) were stained with H&E or PIMO. Tumor necrosis (n represents at least 5) and hypoxia (n represents at least 4) were measured. Bars = means ± SD. *, $p < 0.05$; **, $p < 0.01$; ***, $p < 0.001$. Statistical tests: two-tailed Student's *t*-test for C – H, L and M.

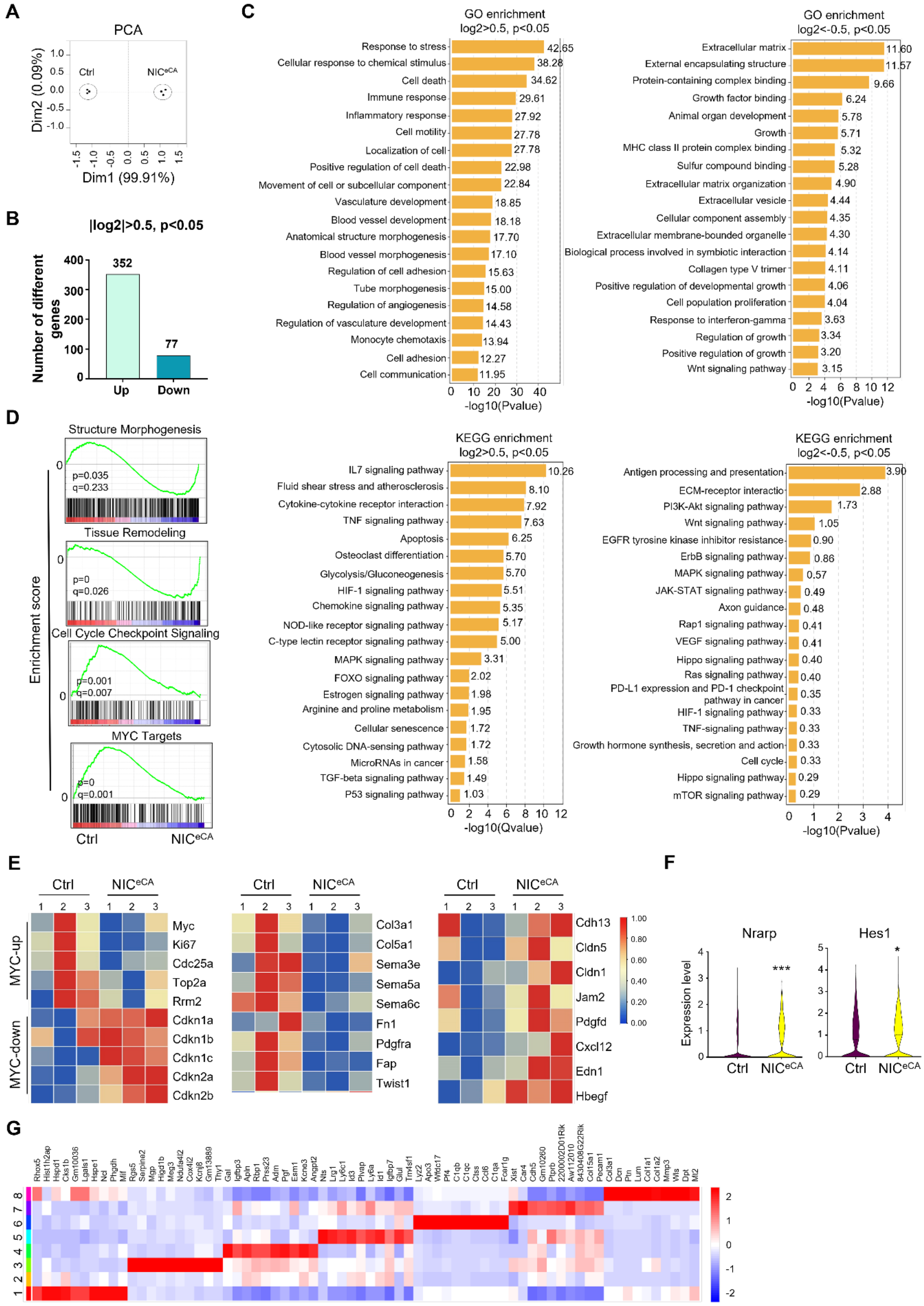

Figure S2. Notch activation downregulates the expression of multiple cell cycle related genes and upregulates the expression of structure morphogenesis related genes. (A)

TECs isolated from Ctrl and NIC^{eCA} mice were subjected to RNA-seq. Principal component analysis (PCA) was conducted between Ctrl and NIC^{eCA} groups. (B) Histogram showed the number of differentially changed genes from (A). (C) The differentially changed genes were subjected to GO and KEGG analysis. Top 20 items were displayed. (D) GSEA analysis of the structure morphogenesis, tissue remodeling, cell cycle checkpoint signaling and MYC targets between Ctrl and NIC^{eCA} mice. (E) Heatmaps of the expression of genes involved proliferation, ECM modification and cell junction and pericyte/vSMC recruitment between NIC^{eCA} and Ctrl mice. (F) TECs isolated from Ctrl and NIC^{eCA} mice were subjected to scRNA-seq. Violin plot showing expression of Notch downstream molecules between NIC^{eCA} and Ctrl groups. (G) Top 10 highly expressed genes from (F) in each cluster of TECs were shown by a heatmap. Bars = means ± SD. *, $p < 0.05$; ***, $p < 0.001$. Statistical tests: two-tailed Student's *t*-test for F.

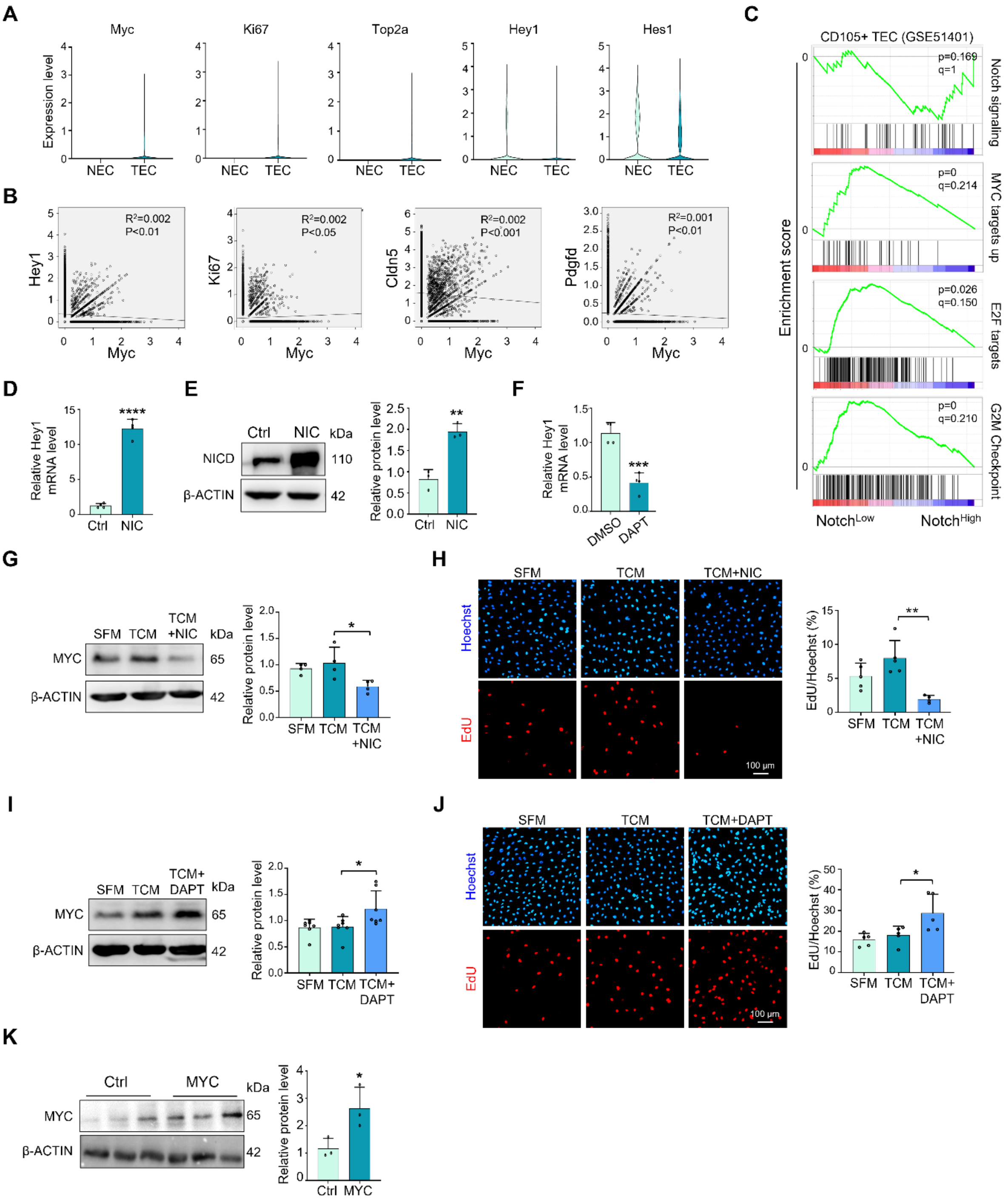

Figure S3. Notch activation inhibits EC proliferation via downregulating MYC expression. (A) Analysis of the published data from reference [1], the expression level of Myc, Ki67, Top2a, Hey1 and Hes1 was displayed and compared between normal ECs (NEC) and tumor ECs (TEC). (B) Analysis of the published data in (A), and the correlation of Myc with Hey1, Ki67, Cldn5, and Pdgfd was determined. (C) GSEA analysis of Notch signaling, MYC targets, E2F targets and G2/M checkpoint between Notch^{High} and Notch^{Low} TECs in

hepatocellular carcinoma (HCC) from the published data (GSE51401). Notch signal degree was determined by the Hey1 expression level. (D and E) HUVECs were infected with adenovirus expressing NIC or control for 24 h. Hey1 (D) and NICD (E) expression levels were determined by qRT-PCR (n = 4) and western blotting (n = 3), respectively. (F) HUVECs were treated with DAPT or DMSO for 24 h. Hey1 expression level was measured by qRT-PCR (n = 4). (G) HUVECs were infected with adenovirus expressing NIC or control, and cultured under SFM or TCM for 24 h. MYC protein level was determined by western blotting (n = 4). (H) HUVECs were treated as in (G). Cell proliferation was evaluated by EdU incorporation assay (n = 5). (I) HUVECs were treated with DAPT or DMSO, and cultured under SFM or TCM for 24 h. MYC protein level was determined by western blotting (n = 7). (J) HUVECs were treated as in (I). Cell proliferation was evaluated by EdU incorporation assay (n = 5). (K) HUVECs were infected with adenovirus expressing MYC or control for 48 h. MYC protein level was determined by western blotting (n = 3). Bars = means ± SD. *, $p < 0.05$; **, $p < 0.01$; ***, $p < 0.001$; ****, $p < 0.0001$. Statistical tests: two-tailed Student's *t*-test for D – F, K; one-way ANOVA followed by Tukey's post hoc test for G - J.

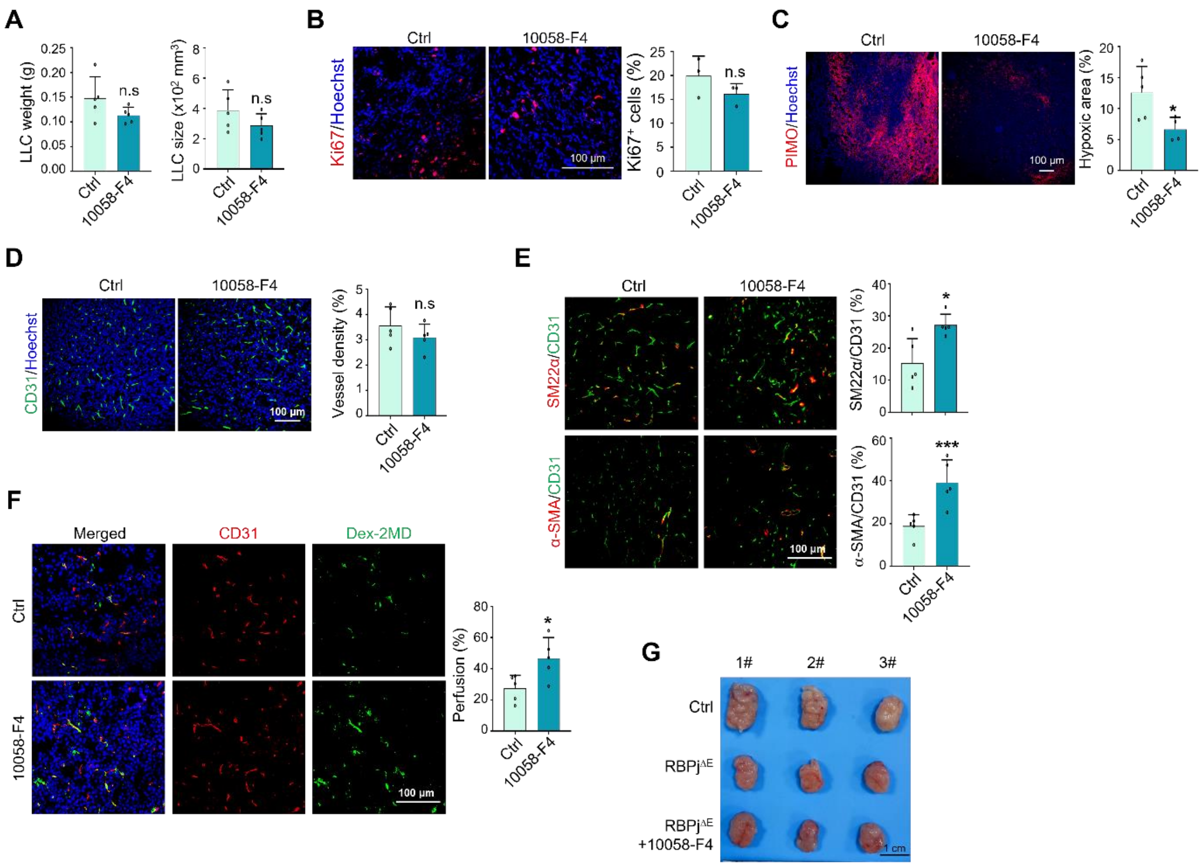

Figure S4. MYC blockade using inhibitor promotes tumor vessel normalization. (A) Mice bearing with LLC were treated with 10058-F4 (30 mg/kg) or DMSO for 14 days. Tumor weight and size were measured and compared (n = 5). (B) Tumor sections from (A) were staining with Ki67 immunofluorescence. Tumor cell proliferation was evaluated (n = 3). (C) Tumor sections from (A) were stained with PIMO immunofluorescence. Tumor hypoxia was determined (n = 5 for Ctrl, n = 4 for 10058-F4). (D) Tumor sections from (A) were stained with CD31 immunofluorescence. Vessel density was quantitatively determined (n = 5). (E) Tumor sections from (A) were stained with CD31 plus α-SMA or SM22α immunofluorescence. Pericyte/vSMC coverage was quantitatively determined (n = 5). (F) Vessel perfusion was determined and quantified by CD31/Dex-2MD immunofluorescence (n = 5). (G) Representative photos for LLC tumor among RBPj$^{\Delta E}$, RBPj$^{\Delta E}$ plus 10058-F4 and Ctrl mice. Bars = means ± SD. *, $p < 0.05$; ***, $p < 0.001$; n.s, not significant. Statistical tests: two-tailed Student's *t*-test for A - F.

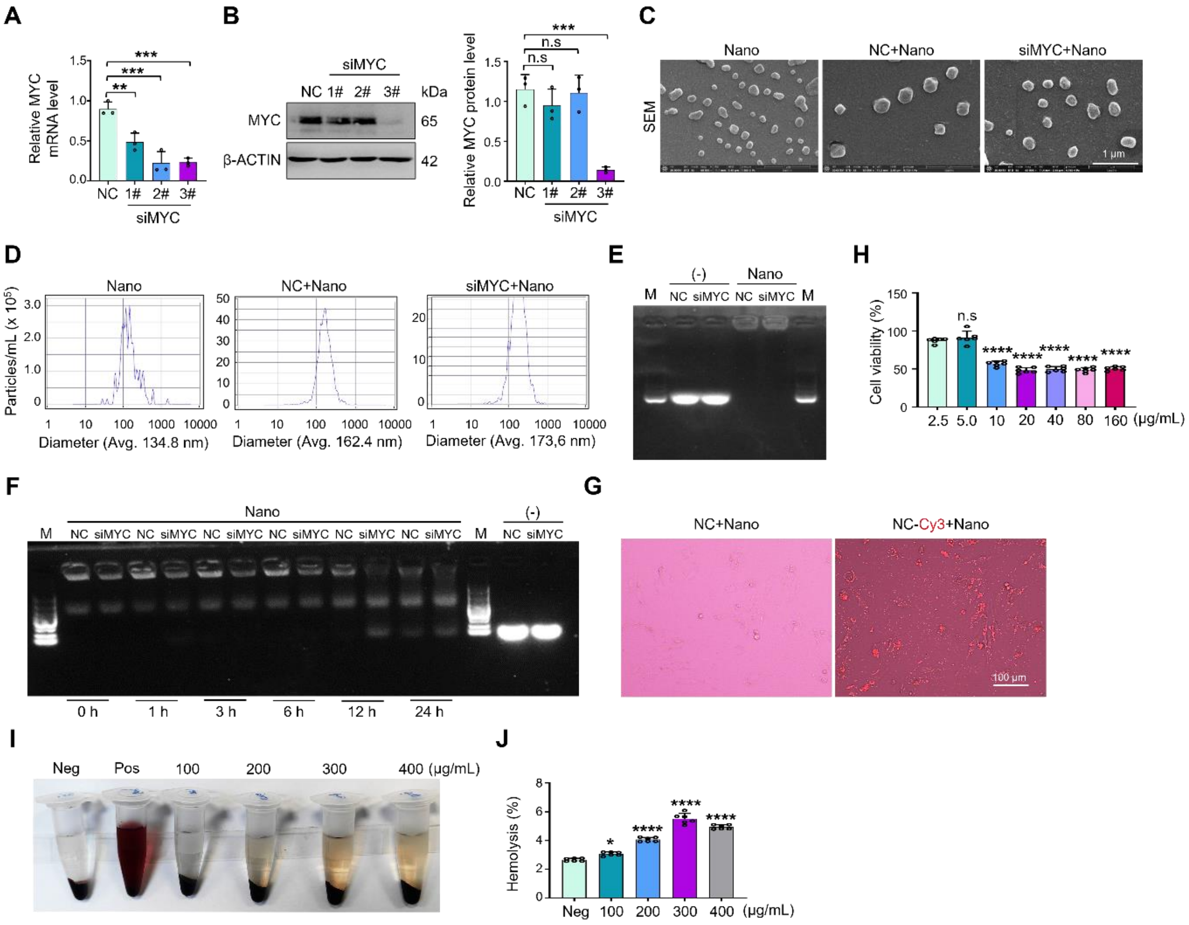

Figure S5. Characterization of PEI-PEG-RGD nanoparticles. (A and B) bEnd.3 cells were transfected with siNC or MYC siRNA for 24 h or 48 h. MYC mRNA and protein levels were determined by qRT-PCR (A) (n = 3) and western blotting (B) (n = 3), respectively. (C) Representative photos of SEM analysis of nanoparticles or nanoparticles encapsulated with NC or siMYC. (D) ZETA analysis of nanoparticles or nanoparticles encapsulated with NC or siMYC. (E) Encapsulation stability of nanoparticles was evaluated by agarose gel retardation assay. (F) Nanoparticles contained siRNA were incubated with 50% mouse serum at 37°C for different time and subjected to electrophoresis. (G) bEnd.3 cells were incubated with nanoparticles encapsulated with Cy3-labeled siRNA. The $Cy3^+$ cells were captured under a fluorescent microscope. (H) bEnd.3 cells were incubated with nanoparticles at different concentration for 12 h. Cell viability was evaluated by CCK8 assay (n = 6). (I and J) Nanoparticles with different concentration were mixed with blood for 1 h. The hemolytic capacity was determined by measuring the supernatant absorbance (n = 6). Bars = means ± SD. *, $p < 0.05$; **, $p < 0.01$; ***, $p < 0.001$; ****, $p < 0.0001$; n.s, not significant. Statistical

tests: one-way ANOVA followed by Tukey's post hoc test for A, B, H, J.

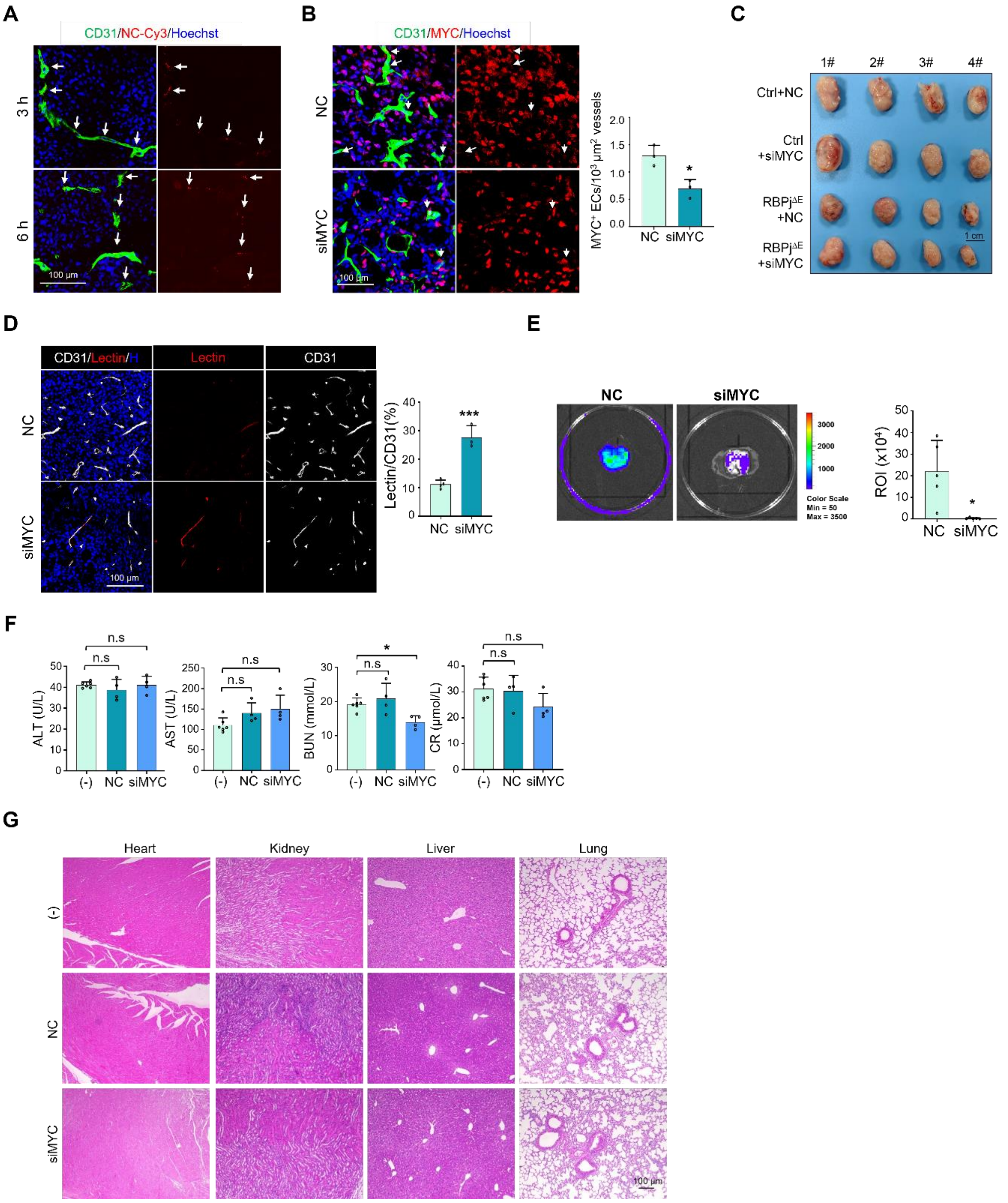

Figure S6. EC-targeted MYC siRNA delivery promotes tumor vessel normalization. (A) Mice inoculated with LLC tumors were administrated with Cy3 labeled siRNA encapsulated with nanoparticles for 3 h and 6 h. The Cy3 positive tumor vessels were determined by immunofluorescence. (B) MYC protein level between siNC and siMYC was determined by immunofluorescence (n = 3). (C) Representative photos for LLC tumors among Ctrl+NC, Ctrl+siMYC, RBPj$^{\Delta E}$+NC, RBPj$^{\Delta E}$+siMYC mice. (D) Mice bearing with LLC tumors were injected with Dylight 594 labeled lectin. Vessel perfusion was determined by

immunofluorescence (n = 4 for NC, n = 3 for siMYC). (E) Mice were inoculated with luciferase^{+} LLC cells and treated with NC or siMYC. Tumors were removed on 14 dpi and mice were further maintained for 21 days. Lungs were evaluated by chemoluminescence (n = 5). (F) Blood biochemistry analysis of the ALT, AST, BUN and CR in serum (n = 5 for (-), n = 4 for NC and siMYC). (G) Tissues of heart, kidney, liver and lung was stained with H&E. Representative photos was captured under a microscope. Bars = means ± SD. *, $p < 0.05$; ***, $p < 0.001$; n.s, not significant. Statistical tests: two-tailed Student's *t*-test for B, D and E; one-way ANOVA followed by Tukey's post hoc test for F. Neg is short for negative. Pos is short for positive. (-) represents no intervention.

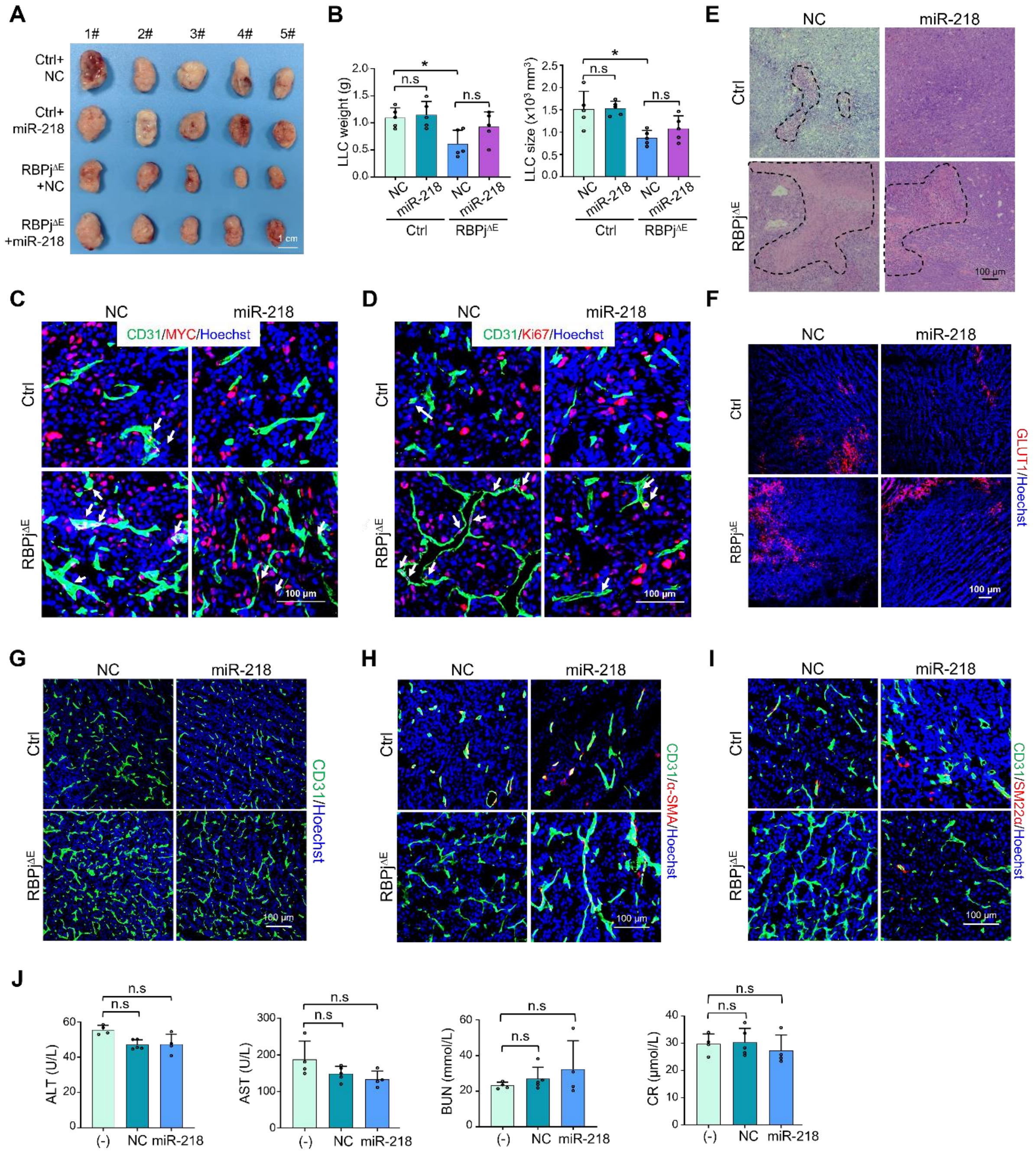

Figure S7. EC-targeted delivery of miR-218 inhibits MYC expression and normalizes tumor vessels. (A and B) RBPj$^{\Delta E}$ and Ctrl mice bearing LLC tumors was intravenously injected with EC-miR-218 or EC-NC. Representative photo for LLC tumors was captured under a camera (A). Tumor weight and size were measured and compared (n = 5) (B). (C) Representative images for tumor sections stained with CD31 plus MYC immunofluorescence. MYC positive TECs were indicated by white arrows. (D) Representative images for tumor sections stained with CD31 plus Ki67 immunofluorescence. Ki67 positive TECs were

indicated by white arrows. (E) Representative images for tumor sections stained with H&E. (F) Representative images for tumor sections stained with GLUT1. (G) Representative images for tumor sections stained with CD31 immunofluorescence. (H) Representative images for tumor sections stained with CD31 plus α-SMA immunofluorescence. (I) Representative images for tumor sections stained with CD31 plus SM22α immunofluorescence. (J) Blood biochemistry analysis of the ALT, AST, BUN and CR in serum from EC-NC or EC-miR-218 treated mice (n = 5 for (-), n = 4 for NC and miR-218). Bars = means ± SD. *, $p < 0.05$; n.s, not significant. Statistical tests: One-way ANOVA followed by Tukey's post hoc test for B and J. (-) represents no intervention.

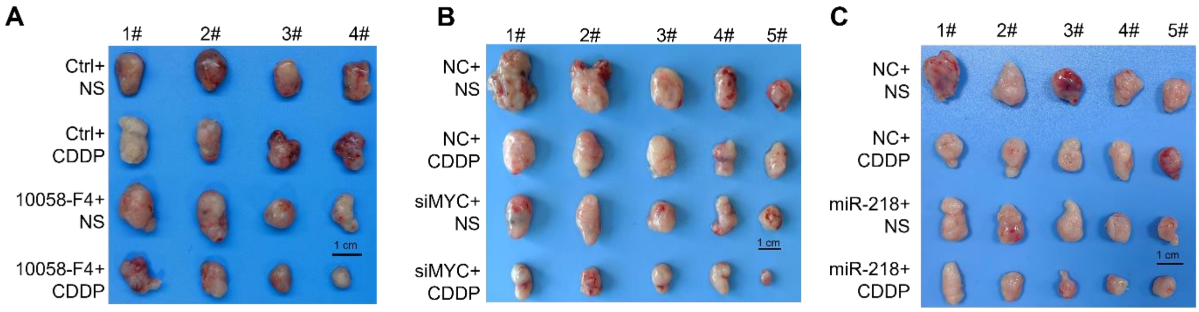

Figure S8. MYC blockade improves the efficiency of chemotherapy. (A) Mice bearing LLC tumors was treated with 10058-F4 or Ctrl plus NS or CDDP. Representative photo for LLC tumors was captured under a camera. (B) Mice bearing LLC tumors was treated with EC-siMYC or EC-NC plus NS or CDDP. Representative photo for LLC tumors was captured under a camera. (C) Mice bearing LLC tumors were treated with EC-miR-218 or EC-NC plus NS or CDDP. Representative photo for LLC tumors was captured under a camera.

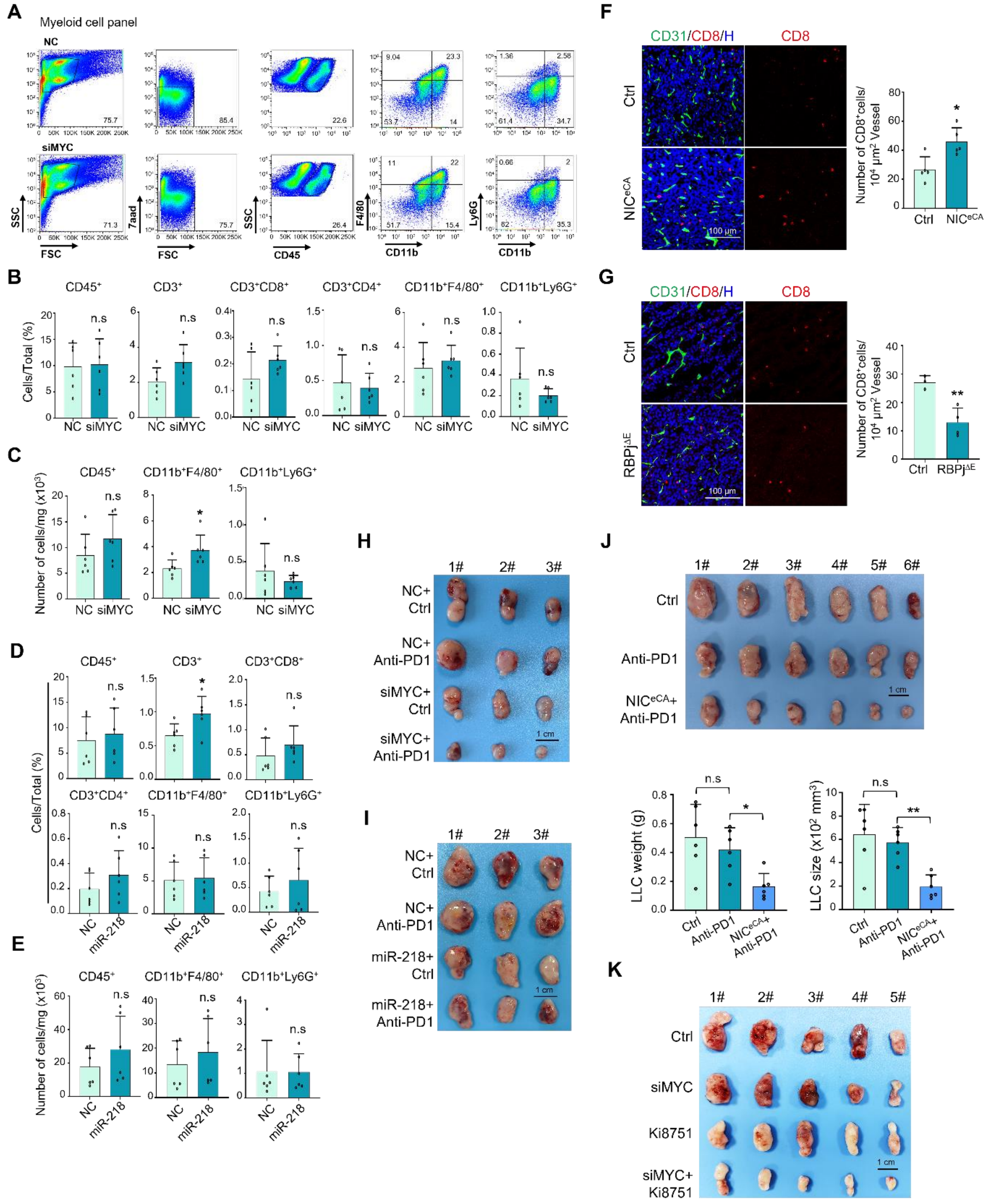

Figure S9. MYC blockade enhances the efficiency of immunotherapy. (A - C) Mice bearing LLC tumors were treated with EC-siMYC or EC-NC for 21 days and subjected to flow cytometry. The percentage (B) and number (C) of T cells and myeloid cells were determined (n = 6). (D and E) Mice bearing LLC tumors were treated with miR-218 or NC nanoparticles for 21 days and subjected to flow cytometry. The percentage (D) and number (E) of T cells and myeloid cells were determined (n = 6). (F) NIC^{eCA} and Ctrl mice were

inoculated with LLC. Tumor sections were stained with CD31 and CD8, and number of $CD8^+$ T cells around per 10^4 μm^2 vessels was counted and compared (n = 5). (G) $RBPj^{\Delta E}$ and Ctrl mice were inoculated with LLC. Tumor sections were stained with CD31 and CD8, and number of $CD8^+$ T cells around per 10^4 μm^2 vessels was counted and compared (n = 3 for Ctrl, n = 4 for $RBPj^{\Delta E}$). (H) Mice bearing LLC tumors were treated with EC-siMYC or EC-NC plus anti-PD1 or Ctrl. Representative photos of tumors were captured under a camera. (I) Mice bearing LLC tumors were treated with EC-miR-218 or EC-NC plus anti-PD1 or Ctrl. Representative photos of tumors were captured under a camera. (J) NIC^{eCA} and Ctrl mice were inoculated with LLC, and treated with saline or anti-PD1 antibody. Photo of tumors was captured under a camera, and tumor weight and size were determined (n = 6). (K) Mice bearing LLC tumors were treated with EC-siMYC or EC-NC plus DMSO or Ki8751. Representative photo for LLC tumors was captured under a camera. Bars = means ± SD. *, $p < 0.05$; **, $p < 0.01$; n.s, not significant. Statistical tests: two-tailed Student's *t*-test for B – G; One-way ANOVA followed by Tukey's post hoc test for J.

References

1. Goveia J, Rohlenova K, Taverna F, Treps L, Conradi LC, Pircher A, et al. An integrated gene expression landscape profiling approach to identify lung tumor endothelial cell heterogeneity and angiogenic candidates. *Cancer Cell*. 2020; 37: 21-36 e13.
